# Increased transport of acetyl‐CoA into the endoplasmic reticulum causes a progeria‐like phenotype

**DOI:** 10.1111/acel.12820

**Published:** 2018-07-27

**Authors:** Yajing Peng, Samantha L. Shapiro, Varuna C. Banduseela, Inca A. Dieterich, Kyle J. Hewitt, Emery H. Bresnick, Guangyao Kong, Jing Zhang, Kathryn L. Schueler, Mark P. Keller, Alan D. Attie, Timothy A. Hacker, Ruth Sullivan, Elle Kielar‐Grevstad, Sebastian I. Arriola Apelo, Dudley W. Lamming, Rozalyn M. Anderson, Luigi Puglielli

**Affiliations:** ^1^ Department of Medicine University of Wisconsin‐Madison Madison Wisconsin; ^2^ Waisman Center University of Wisconsin‐Madison Madison Wisconsin; ^3^ Neuroscience Training Program University of Wisconsin‐Madison Madison Wisconsin; ^4^ Department of Cell and Regenerative Biology University of Wisconsin‐Madison Madison Wisconsin; ^5^ Department of Biochemistry University of Wisconsin‐Madison Madison Wisconsin; ^6^ Cardiovascular Research Center University of Wisconsin‐Madison Madison Wisconsin; ^7^ Department of Comparative Biosciences University of Wisconsin‐Madison Madison Wisconsin; ^8^ Geriatric Research Education Clinical Center Veterans Affairs Medical Center Madison Wisconsin; ^9^ Department of Neuroscience University of Wisconsin‐Madison Madison Wisconsin; ^10^ Present address: Department of Internal Medicine University of Michigan Ann Arbor Michigan; ^11^ Present address: Department of Dairy Science University of Wisconsin‐Madison Madison Wisconsin

**Keywords:** acetyl‐CoA, AT‐1/SLC33A1, ATase1, ATase2, lysine acetylation, progeria

## Abstract

The membrane transporter AT‐1/SLC33A1 translocates cytosolic acetyl‐CoA into the lumen of the endoplasmic reticulum (ER), participating in quality control mechanisms within the secretory pathway. Mutations and duplication events in *AT‐1/SLC33A1* are highly pleiotropic and have been linked to diseases such as spastic paraplegia, developmental delay, autism spectrum disorder, intellectual disability, propensity to seizures, and dysmorphism. Despite these known associations, the biology of this key transporter is only beginning to be uncovered. Here, we show that systemic overexpression of AT‐1 in the mouse leads to a segmental form of progeria with dysmorphism and metabolic alterations. The phenotype includes delayed growth, short lifespan, alopecia, skin lesions, rectal prolapse, osteoporosis, cardiomegaly, muscle atrophy, reduced fertility, and anemia. In terms of homeostasis, the AT‐1 overexpressing mouse displays hypocholesterolemia, altered glycemia, and increased indices of systemic inflammation. Mechanistically, the phenotype is caused by a block in Atg9a‐Fam134b‐LC3β and Atg9a‐Sec62‐LC3β interactions, and defective reticulophagy, the autophagic recycling of the ER. Inhibition of ATase1/ATase2 acetyltransferase enzymes downstream of AT‐1 restores reticulophagy and rescues the phenotype of the animals. These data suggest that inappropriately elevated acetyl‐CoA flux into the ER directly induces defects in autophagy and recycling of subcellular structures and that this diversion of acetyl‐CoA from cytosol to ER is causal in the progeria phenotype. Collectively, these data establish the cytosol‐to‐ER flux of acetyl‐CoA as a novel event that dictates the pace of aging phenotypes and identify intracellular acetyl‐CoA‐dependent homeostatic mechanisms linked to metabolism and inflammation.

## 1 INTRODUCTION

Nε‐lysine acetylation in the lumen of the endoplasmic reticulum (ER) has emerged as a novel mechanism for the regulation of protein homeostasis (also referred to as proteostasis) within the organelle (Ding, Dellisanti, Ko, Czajkowski, & Puglielli, 2014; Hullinger et al., 2016; Jonas, Pehar, & Puglielli, 2010; Pehar, Jonas, Hare, & Puglielli, 2012; Peng & Puglielli, 2016; Peng et al., 2016, 2014). Acetylation of ER cargo proteins is ensured by three essential elements: AT‐1, ATase1, and ATase2. AT‐1 (also referred to as SLC33A1) is the ER membrane transporter that regulates the cytosol‐to‐ER flux of acetyl‐CoA, donor of the acetyl group in the reaction of Nε‐lysine acetylation (Jonas et al., 2010; Peng et al., 2014). ATase1 (also referred to as NAT8B) and ATase2 (also referred to as NAT8) are type II ER membrane proteins that carry out the reaction of Nε‐lysine acetylation within the ER lumen (Ding et al., 2014; Ko & Puglielli, 2009). Ex vivo and in vivo data suggest that the ER acetylation machinery is a component of ER quality control and regulates two essential functions of the organelle: (a) selection and transport of cargo proteins along the secretory pathway; and (b) disposal of protein aggregates that form within the ER and secretory pathway (Ding et al., 2014; Hullinger et al., 2016; Pehar et al., 2012; Peng & Puglielli, 2016; Peng et al., 2016, 2014). The former requires the ATases to associate with the oligosaccharyltransferase complex (OST) and acetylate correctly folded polypeptides (Ding et al., 2014; Peng & Puglielli, 2016) while the latter requires acetylation/deacetylation of the autophagy protein Atg9a (Pehar et al., 2012; Peng & Puglielli, 2016; Peng et al., 2016, 2014 ). AT‐1 regulates availability of acetyl‐CoA within the ER; as such, it plays crucial regulatory functions for the entire ER acetylation machinery.

Clinically, diverse outcomes related to genetic deficiency, mutation, or overabundance of AT‐1 have been identified. Children with homozygous mutations in *AT‐1/SLC33A1* display congenital defects, severe developmental delay, and premature death (Chiplunkar et al., 2016; Huppke et al., 2012). Patients with heterozygous mutations appear normal at birth but then develop a complicated autosomal dominant form of spastic paraplegia (Lin et al., 2008). Finally, chromosomal duplications of the 3q25.31 locus, which harbors *AT‐1/SLC33A1*, have been reported in patients with autism spectrum disorder (ASD), intellectual disability, propensity to seizures, and facial dysmorphism (SFARI database; see also Swisshelm et al. 2014. ASHG Annual Meeting; Abstract 3205 T). The above disease phenotypes are mimicked by related mouse models. Knock‐in mice that lack AT‐1 activity (AT‐1^S113R/S113R^) die during embryogenesis, while mice with haploinsufficiency of AT‐1 (AT‐1^S113R/+^) develop neurodegeneration with spasticity and propensity to infections and cancer (Peng et al., 2014). Transgenic mice that overexpress AT‐1 in forebrain neurons (AT‐1 Tg) display an ASD‐like phenotype without dysmorphism (Hullinger et al., 2016). Mechanistically, the phenotype of AT‐1^S113R/+^ mice is linked to aberrant activation of autophagy (Peng et al., 2014) while the phenotype of AT‐1 Tg is linked to increased efficiency of the secretory pathway (Hullinger et al., 2016). When taken together, the convergence of human‐ and mouse‐based studies clearly indicates that the ER acetylation machinery plays fundamental biological functions.

Despite these known associations, the biology of AT‐1 and the ER acetylation machinery is only beginning to be uncovered. Here, we sought to investigate the broader consequences of AT‐1 manipulation, including the cellular and systemic impact of AT‐1‐directed changes in ER acetylation, by generating a transgenic mouse where AT‐1 was placed under the control of the Rosa26 locus. The animals developed a severe phenotype mimicking segmental forms of human progerias. At a mechanistic level, we observed increased acetylation of the ER‐localized autophagy protein Atg9a, reduced Atg9a‐Fam134b‐LC3β and Atg9a‐Sec62‐LC3β interaction, and a block in ER‐autophagy/reticulophagy. Finally, we showed that a specific ATase1/ATase2 inhibitor, which restores ER proteostatic functions downstream of AT‐1, was able to rescue the entire phenotype of the animals, including the lifespan. Collectively, these data establish the cytosol‐to‐ER flux of acetyl‐CoA as a novel event that dictates the pace of aging phenotypes and identify intracellular acetyl‐CoA‐dependent homeostatic mechanisms linked to metabolism and inflammation.

## 2 RESULTS

### 2.1 AT‐1 sTg mice display a progeria‐like phenotype

**Figure 1:**
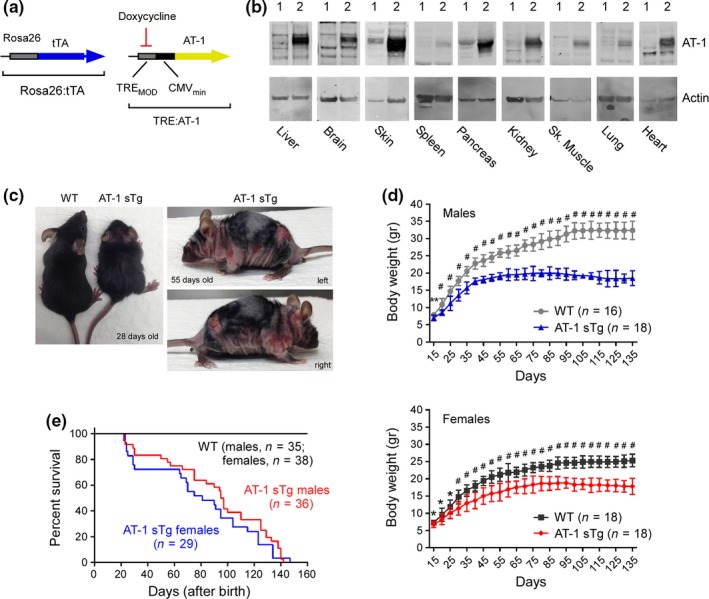
AT‐1 sTg mice are smaller and have a short lifespan. (a) AT‐1 sTg mice were generated with an inducible Tet‐Off expression system under the control of the Rosa26 locus for systemic overexpression. (b) Western blots showing AT‐1 overexpression in different tissues (*1*, WT; *2*, AT‐1 sTg). (c) Representative AT‐1 sTg mouse and WT littermate when 28 and 55 days old. (d) Body weight of male and female WT and AT‐1 sTg mice across their lifespan. (e) Lifespan of AT‐1 sTg mice (*maximum lifespan*, males = 142 days, females = 147 days, *p* < 0.0005; *median lifespan*, males = 96 days, females = 81 days, *p* < 0.0005). Bars represent mean ± *SD*. ^*^ *p* < 0.05; ^**^ *p* < 0.005; ^#^ *p* < 0.0005

To study the systemic role of AT‐1, we generated transgenic (Tg) mice with an inducible overexpression Tet‐Off system driven by the Rosa26 locus (Figure 1a,b). For the purpose of this study, the animals (referred to as AT‐1 sTg) were maintained in the absence of doxycycline (Dox); therefore, they overexpressed AT‐1 throughout their entire life, including development. The animals were born with Mendelian ratio and were completely normal at birth. However, within 1 month they appeared smaller than their wild‐type/non‐Tg (WT) littermates (Figure 1c), and within 2 months they displayed a severe phenotype (Figure 1c and Table 1) that was reminiscent of segmental forms of human progerias (Gonzalo, Kreienkamp, & Askjaer, 2017; Karikkineth, Scheibye‐Knudsen, Fivenson, Croteau, & Bohr, 2017; Liao & Kennedy, 2014; Pivnick et al., 2000). Indeed, they remained smaller throughout their entire lifespan (Figure 1d), appeared phenotypically old (Figure 1c), and displayed a very short lifespan (Figure 1e). The progeria‐like features of the animals are listed in Table 1.

**Table 1:** Observed phenotype of AT‐1 sTg mice

Median lifespan	Greatly reduced (males, 96 days; females, 81 days)
Maximum lifespan	Greatly reduced (males, 142 days; females, 147 days)
Body weight	Reduced
Dysmorphism	Observed
Major organ mass	Increased (exception: uterus, decreased)
Fertility	Males, normal; females, reduced
Adipose tissue	Reduced
Lordokyphosis	Modest or absent
Cardiomegaly	Pronounced
Osteoporosis	Pronounced
Hair loss	Pronounced
Hair regrowth	Greatly reduced
Skin lesions	Pronounced
Wound repair	Retarded
Muscle atrophy	Modest to severe
Cataracts	Normal (as in WT)
Dermal thickness	Reduced
Rectal prolapse	Common
Systemic inflammation	Pronounced
Peripheral WBC	Altered (B cell and neutrophil expansion)
Peripheral RBC	Altered (anemia)
Serum glucose/insulin	Reduced
Serum lipids	Hypocholesterolemia
Glucose tolerance	Altered

**Figure 2:**
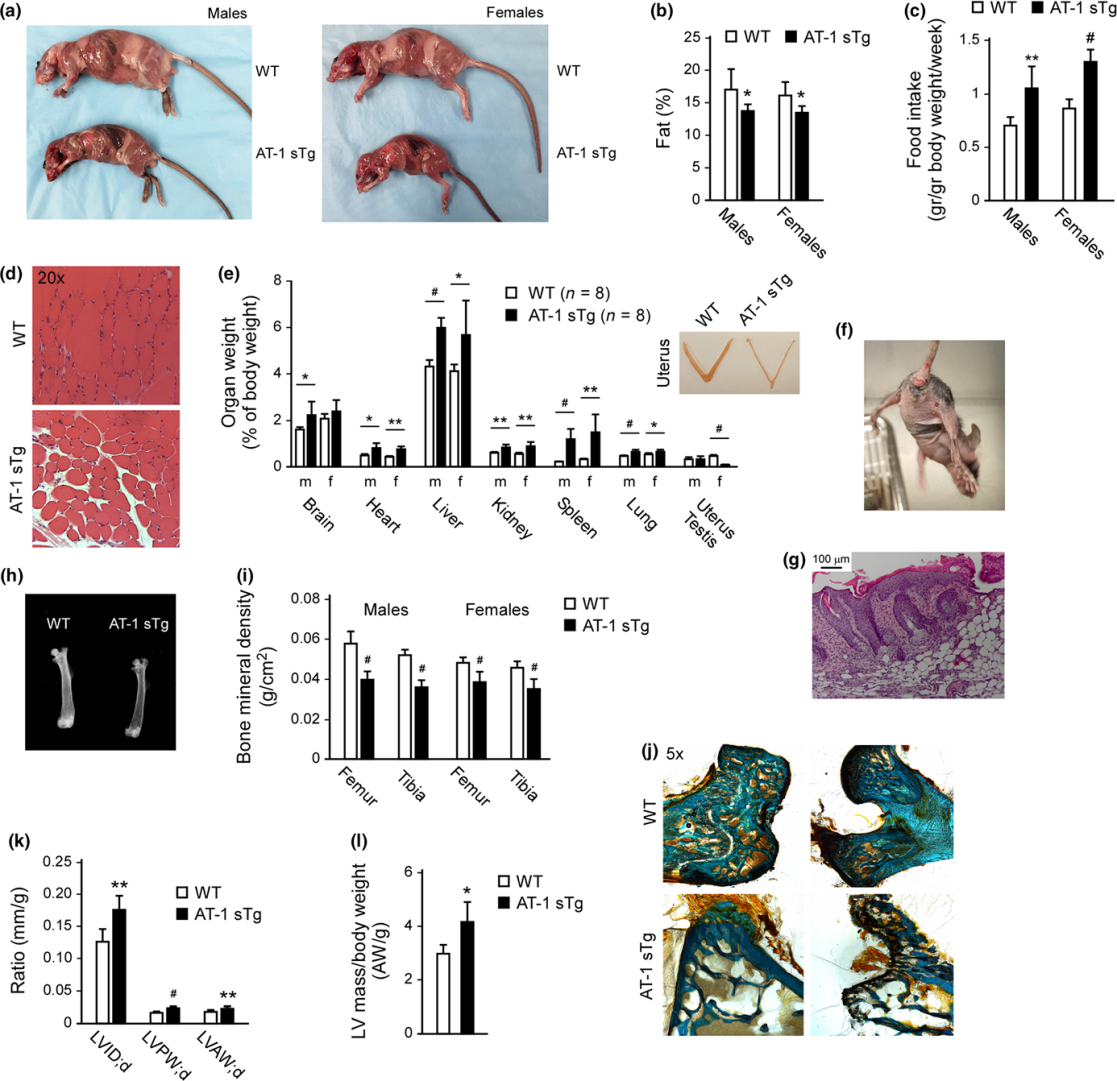
AT‐1 sTg mice display progeria‐like features. (a) Examination of WT and AT‐1 sTg mice. Reduced fat accumulation and splenomegaly are evident in both AT‐1 sTg males and females. (b) Total body fat in WT and AT‐1 sTg mice as determined by dual‐energy X‐ray (DEXA) scanning (males, *n* = 7; females, *n* = 8). (c) Food intake of WT and AT‐1 sTg mice (males, *n* = 5; females, *n* = 5). (d) Skeletal muscle histology. (e) Weight of major organs. *Inset* shows uterus. (f) Skin alterations and rectal prolapse in AT‐1 sTg mice. (g) H&E staining of a skin section from AT‐1 sTg mice. (h, i) Faxitron X‐ray (femur) (h) and bone mineral density (i) of WT and AT‐1 sTg mice (WT, *n* = 8; AT‐1 sTg, *n* = 8). (j) Goldner's trichrome stain of femur sections. (k, l) Echocardiographic assessment of WT and AT‐1 sTg mice (WT, *n* = 8; AT‐1 sTg, *n* = 8). Bars represent mean ± *SD*. ^*^ *p* < 0.05, ^**^ *p* < 0.005, ^#^ *p* < 0.0005. LVID*: d*, left ventricular internal diameter end diastole; LVPW*: d*, left ventricular posterior wall end diastole; LVAW*: d*, left ventricular anterior wall end diastole

In addition to being small, AT‐1 sTg mice were very thin and had small fat pads (Figure 2a,b); the significant reduction in fat tissue was observed despite the fact that they ate more than their WT littermates (Figure 2c). AT‐1 sTg mice also displayed small areas of muscle atrophy (Figure 2d). However, relative to their body weight, they had overall enlarged organs (Figure 2e); the only exception was the uterus, which appeared significantly smaller and atrophic (Figure 2e; see Inset). This finding is in line with the observed reduced fertility of female animals (Table 1). The skin displayed hair loss, multiple lesions, and delayed wound repair (Figures 1c and 2f; Table 1). Histologically, we observed dermatitis with marked acanthosis and moderate orthokeratotic hyperkeratosis, reactive fibrosis, and epidermal hyperplasia (Figure 2g). Most of the animals also developed rectal prolapse (Figure 2f). Both male and female AT‐1 sTg mice displayed severe bone density loss, which was reminiscent of human osteoporosis (Figure 2h–j). In line with the postmortem data (Figure 2e), echocardiographic assessment of living animals confirmed that AT‐1 sTg mice suffered from cardiomegaly very early in life (Figure 2k,l).

### 2.2 AT‐1 sTg mice display defective hematopoiesis, metabolic alterations, and systemic inflammation

**Figure 3:**
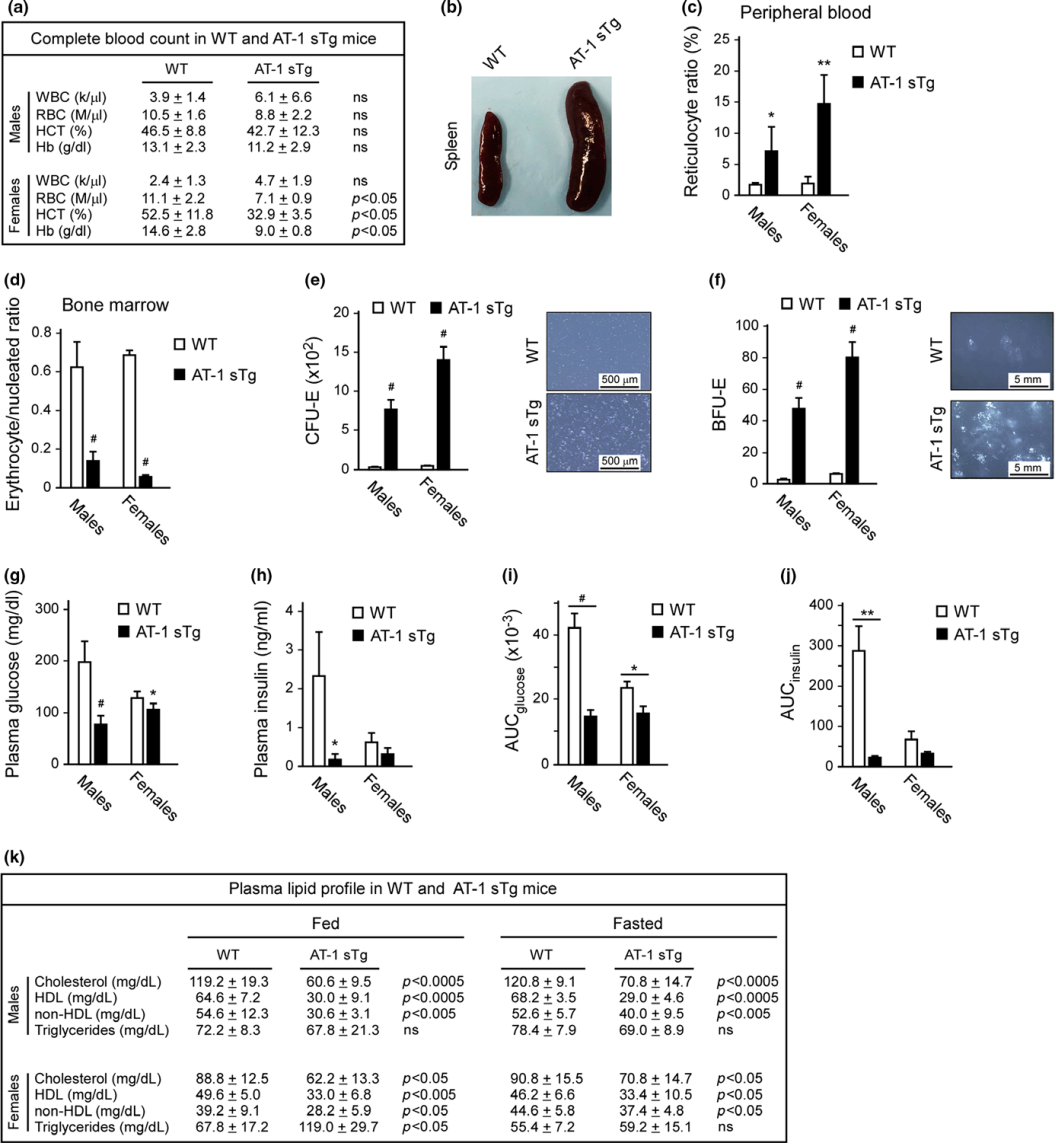
AT‐1 sTg mice display defective hematopoiesis, and reduced levels of circulating glucose, insulin, and cholesterol. (a) Hematologic parameters of WT and AT‐1 sTg mice (males, *n* = 4; females, *n* = 6). (b) Representative images of whole spleen from WT and AT‐1 sTg mice. (c, d) Quantitative blood (c) and bone marrow (d) smears in WT and AT‐1 sTg mice (males, *n* = 4; females, *n* = 4). (e) Quantitation (left) and representative images (right) of colony‐forming unit‐erythroid (CFU‐E) colonies 2 days after plating splenic cells (1 × 10^5^) from WT (*n* = 3) and AT‐1 sTg (*n* = 3) mice in methylcellulose containing Epo, SCF, IL‐3, and IL‐6. (f) Quantitation (left) and representative images (right) of burst‐forming unit‐erythroid (BFU‐E) colonies 5 days after plating splenic cells (1 × 10^5^) from WT (*n* = 3) and AT‐1 sTg (*n* = 3) mice in methylcellulose containing Epo, SCF, IL‐3, and IL‐6. (g, h) Fasting levels of glucose (g) and insulin (h) in plasma (males, *n* = 5; females, *n* = 5). (i, j) Oral glucose tolerance test (OGTT) in WT and AT‐1 sTg mice (males, *n* = 5; females, *n* = 5). AUC_glucose_ (i) and AUC_insulin_ (j) are shown. (k) Plasma lipid profile in WT and AT‐1 sTg mice (males, *n* = 5; females, *n* = 5). Bars represent mean ± *SD*. ^*^ *p* < 0.05, ^**^ *p* < 0.005, ^#^ *p* < 0.0005. WBC*:* white blood cells; RBC: red blood cells; HCT: hematocrit; Hb: hemoglobin. WBC*:* white blood cells; RBC: red blood cells; HCT: hematocrit; Hb: hemoglobin

A complete blood count of AT‐1 sTg mice revealed mild‐to‐moderate anemia, which was well evident in females (Figure 3a). The anemia was accompanied by splenomegaly and expansion of the spleen interfollicular/red pulp (extramedullary hematopoiesis) in both males and females (Figure 3b). We also observed reduced levels of circulating ferritin (Supporting Information Figure S1a) and iron (Supporting Information Figure S1b), increased reticulocyte ratio in the peripheral blood (Figure 3c), and reduced erythrocyte/nucleated ratio in the bone marrow (Figure 3d). Further assessment revealed a marked increase in erythroid progenitor activity in AT‐1 sTg spleens relative to WT (Figure 3e,f). When taken together, the above results indicate that AT‐1 overexpression disrupts steady‐state hematopoiesis, causing splenomegaly and extramedullary erythropoiesis.

Metabolic assessment of AT‐1 sTg mice revealed reduced levels of circulating glucose and insulin (Figure 3g,h) but normal levels of glucagon (Supporting Information Figure S2a). Lower levels of plasma glucose and insulin were also observed following an oral glucose tolerance test (Figure 3i,j and Supporting Information Figure S2b,c) suggesting a more effective utilization of glucose. The metabolic assessment of the animals also revealed reduced levels of circulating cholesterol (Figure 3k).

**Figure 4:**
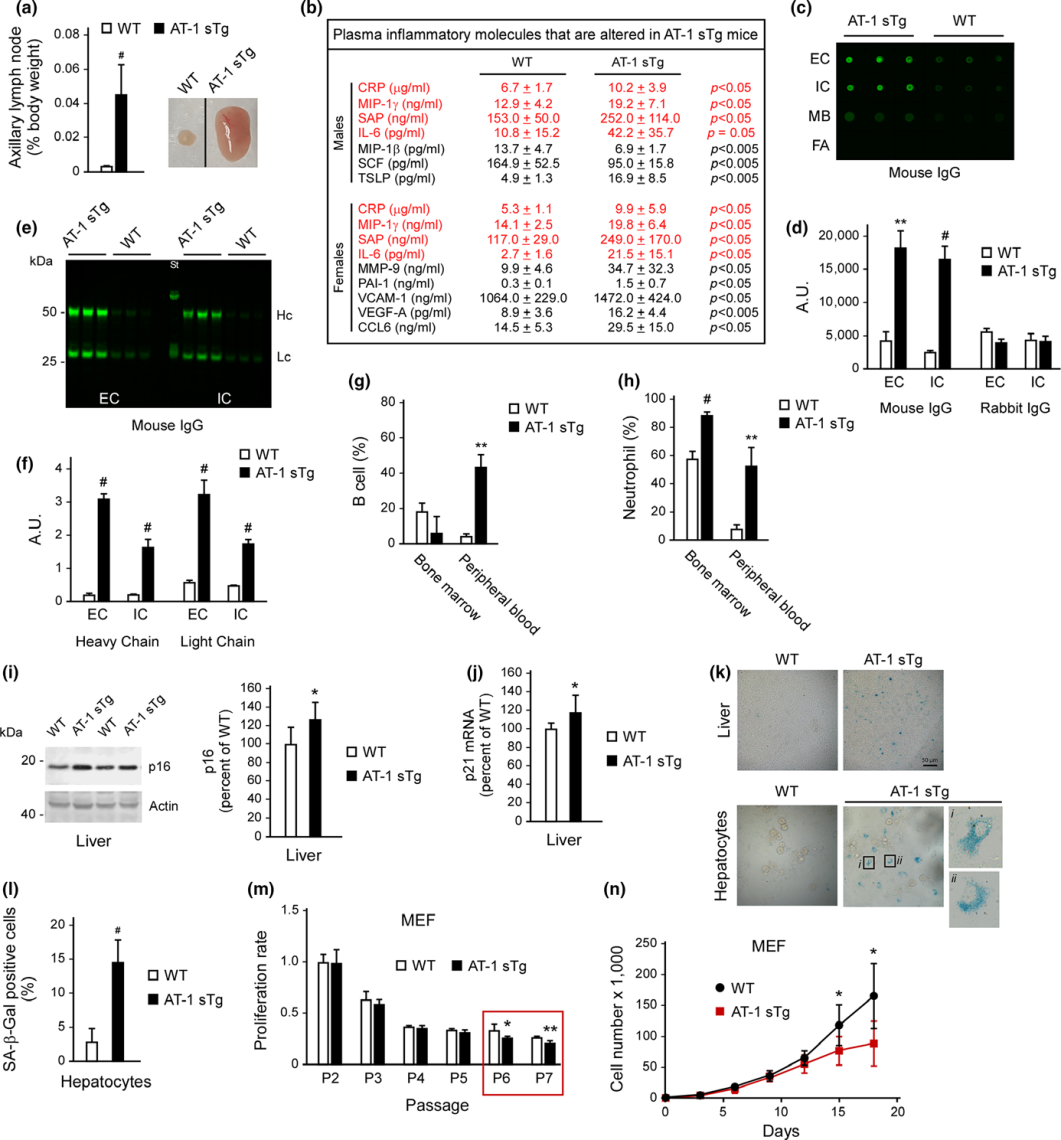
AT‐1 sTg mice display systemic and tissue inflammation. (a) Weight of axillary lymph nodes in WT and AT‐1 sTg mice (*n* = 6 per group). (b) Plasma inflammatory markers (out of a total of 42 different analytes tested). In red, analytes that were changed in both males and females (males, *n* = 6; females, *n* = 6). (c, d) Dot blot of tissue immunoglobulins determined with anti‐mouse IgG. Representative images (c) and quantitation of results (d) are shown. The analysis was carried out in liver (WT, *n* = 3; AT‐1 sTg, *n* = 3). (e, f) Western blot showing tissue immunoglobulins determined with anti‐mouse IgG. Representative images (e) and quantitation of results (f) are shown. The analysis was carried out in liver (WT, *n* = 3; AT‐1 sTg, *n* = 3). (g, h) Flow cytometry showing B cell (g), and neutrophil (h) population in bone marrow and peripheral blood (WT, *n* = 3; AT‐1 sTg, *n* = 3). (i) Western blot showing p16 levels in liver. Representative images (left panel) and quantitation of results (right panel) are shown (WT, *n* = 7; AT‐1 sTg, *n* = 7). (j) p21 mRNA quantitation in liver (WT, *n* = 7; AT‐1 sTg, *n* = 7). (k, l) SA‐β‐Gal staining in liver and hepatocytes. Representative images (k) and quantitation of results (l) are shown (WT, *n* = 3; AT‐1 sTg, *n* = 3). (m, n) Proliferation potential of cultured MEF expressed as proliferation rate at each passage (m; *n* = 3 different MEF lines/group) and as cell number after plating (n; *n* = 3 different MEF lines/group). Bars represent mean ± *SD*. ^*^ *p* < 0.05, ^**^ *p* < 0.005, ^#^ *p* < 0.0005. EC: extracellular; FA: formic acid‐soluble; Hc: heavy chain; IC: intracellular; Lc: light chain; MB: membrane‐bound

Postmortem examination of AT‐1 sTg mice revealed enlarged lymph nodes (Figure 4a) as well as histological evidence for moderate inflammatory infiltration across different tissues and organs, indicative of systemic inflammation. Consistently, we observed increased levels of several inflammatory molecules in the plasma (Figure 4b) as well as a marked immunoglobulin infiltration of peripheral tissues (Figure 4c–f). The increased systemic inflammation in AT‐1 sTg mice was also reflected in the significant increase in B cells and neutrophils in the peripheral blood relative to WT (Figure 4g,h). Chronic tissue inflammation is often associated with markers of cellular senescence (Jeon et al., 2017; Kang et al., 2015; Ovadya & Krizhanovsky, 2014; Tchkonia, Zhu, Deursen, Campisi, & Kirkland, 2013). Therefore, we used isolated hepatocytes and liver sections to determine levels of p16, p21, and senescence‐associated β‐galactosidase (SA‐β‐Gal), three established markers of cell senescence (Jeon et al., 2017; Kang et al., 2015; Ovadya & Krizhanovsky, 2014; Tchkonia et al., 2013). We consistently found increased levels of the senescent markers in AT‐1 sTg mice when compared to WT littermates (Figure 4i–l). These results were paralleled by a significant reduction in the proliferation potential of mouse embryonic fibroblasts (MEF) in culture (Figure 4m,n).

### 2.3 AT‐1 sTg mice display defective reticulophagy

The above results indicate that systemic overexpression of AT‐1 causes a complex phenotype that resembles human segmental progerias with metabolic alterations. We previously reported that the influx of acetyl‐CoA from the cytosol to the ER lumen by AT‐1 regulates the induction of ER‐autophagy and the disposal of protein aggregates within the secretory pathway (Jonas et al., 2010; Pehar et al., 2012; Peng & Puglielli, 2016; Peng et al., 2016, 2014 ). Therefore, it is possible that a defect in ER‐autophagy (also referred to as reticulophagy) is at the basis of the progeria‐like phenotype of AT‐1 sTg mice.

**Figure 5:**
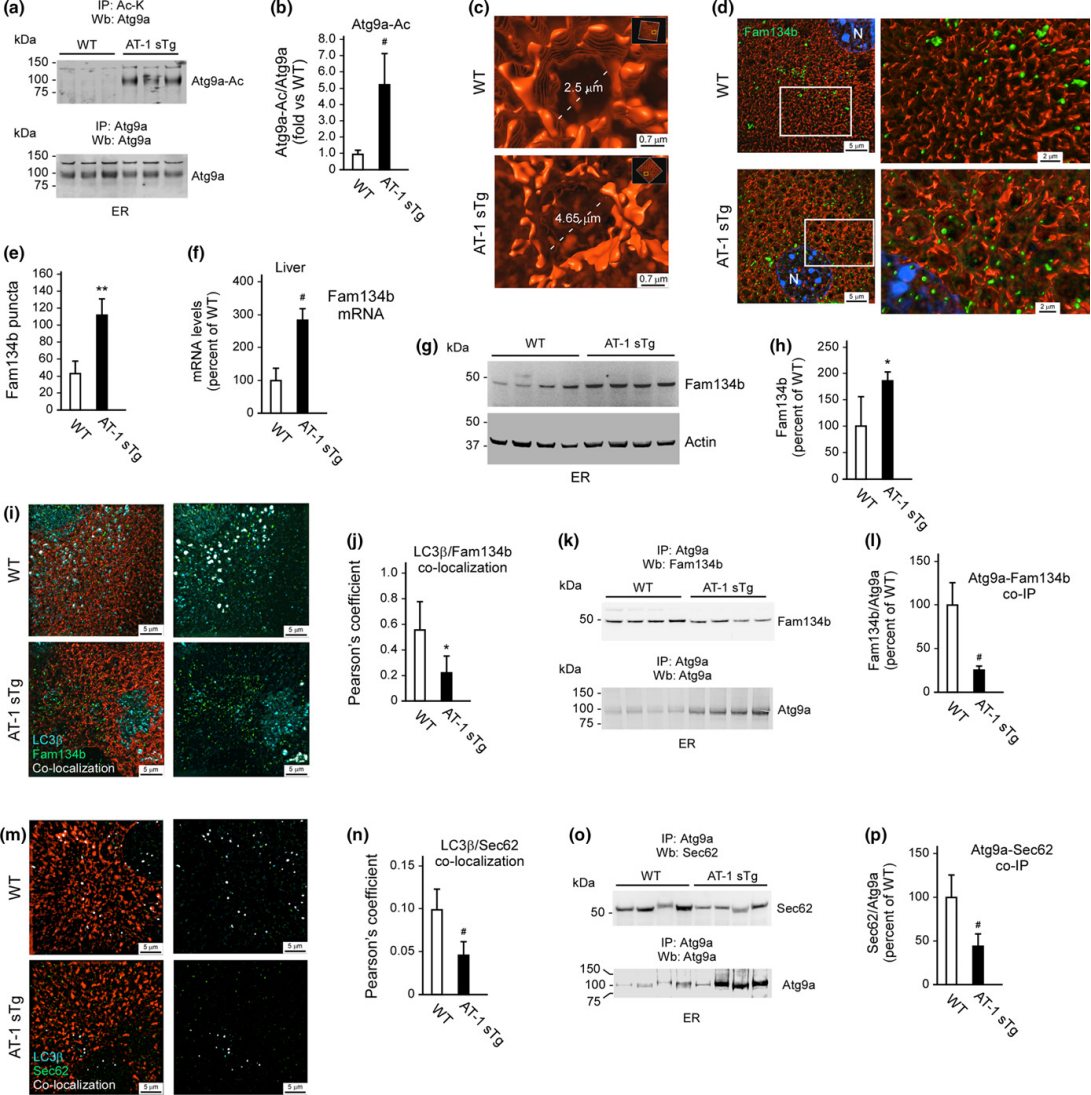
AT‐1 sTg mice display defective reticulophagy and expansion of the ER. (a, b) Western blot showing levels of acetylated‐Atg9a (Atg9a‐Ac) in ER preparations from liver. Representative blots are shown in (a) while quantitation of results is shown in (b) (WT, *n* = 3; AT‐1 sTg, *n* = 3). (c) Structure illumination microscopy (SIM) of ER in isolated hepatocytes showing size and morphology of sheet‐like structures. (d, e) SIM showing Fam134b puncta on ER of isolated hepatocytes. Quantitation of results is shown in (e) (WT, *n* = 8; AT‐1 sTg, *n* = 7). (f) Fam134b mRNA quantitation in liver (WT, *n* = 6; AT‐1 sTg, *n* = 5). (g, h) Western blot showing levels of Fam134b in ER preparations. Representative blots are shown in (g) while quantitation of results is shown in (h) (WT, *n* = 4; AT‐1 sTg, *n* = 4). (i, j) SIM showing reduced Fam134b/LC3β co‐localization on ER of isolated hepatocytes from AT‐1 sTg mice. Quantitation of results is shown in (j) (WT, *n* = 5; AT‐1 sTg, *n* = 5). (k, l) Western blot showing co‐immunoprecipitation of Atg9a and Fam134b in WT and AT‐1 sTg mice. Representative blots are shown in (k) while quantitation of results is shown in (l) (WT, *n* = 8; AT‐1 sTg, *n* = 8). (m, n) SIM showing reduced Sec62/LC3β co‐localization on ER of isolated hepatocytes from AT‐1 sTg mice. Quantitation of results is shown in (n) (WT, *n* = 5; AT‐1 sTg, *n* = 5). (o, p) Western blot showing co‐immunoprecipitation of Atg9a and Sec62 in WT and AT‐1 sTg mice. Representative blots are shown in (o) while quantitation of results is shown in (p) (WT, *n* = 7; AT‐1 sTg, *n* = 7). Bars represent mean ± *SD*. ^*^ *p* < 0.05, ^**^ *p* < 0.005, ^#^ *p* < 0.0005. N: nucleus

To test the above hypothesis, we first analyzed the acetylation profile of the autophagy protein Atg9a, which is essential for the induction of autophagy downstream of the ER acetylation machinery (Pehar et al., 2012; Peng & Puglielli, 2016; Peng et al., 2016, 2014 ). In fact, Atg9a undergoes acetylation on two lysine residues, K359 and K363, which face the lumen of the ER (Pehar et al., 2012). Acetylated Atg9a blocks the induction of autophagy while nonacetylated Atg9a exerts the opposite effect (Pehar et al., 2012). Direct assessment of ER membranes from WT and AT‐1 sTg mice revealed a marked increase in the acetylation status of Atg9a in the transgenic animals (Figure 5a,b), thus supporting our hypothesis. To assess whether the increased acetylation of Atg9a was accompanied by reduced disposal of misfolded/aggregated ER cargo proteins, we took advantage of the pro‐aggregating properties of the A53 T mutant form of α‐synuclein (A53 T syn; Polymeropoulos et al., 1997). Specifically, we used A53 T syn with a signal peptide (SP) at the N‐terminus to direct translation on the ER and insertion into the secretory pathway (Peng et al., 2016). The results show that when expressed in MEF, the levels of aggregated/SDS soluble SP‐A53 T syn were higher in AT‐1 sTg vs. WT animals (Supporting Information Figure S3a,b). This finding is consistent with previous data, where we showed a more efficient clearance of SP‐A53 T syn in MEF from AT‐1^S113R/+^ mice, which display reduced AT‐1 activity, reduced acetylation of Atg9a, and increased induction of ER‐autophagy (Peng et al., 2016). Therefore, when taken together, data from two different animal models, AT‐1 sTg (present study) and AT‐1^S113R/+^ (Peng et al., 2016) mice, as well as cellular systems (Pehar et al., 2012), support the conclusion that the increased acetylation of Atg9a in AT‐1 sTg mice is causally linked to reduced ability of the ER to dispose of toxic protein aggregates that form within its lumen (see also later).

Previous data in MEF from AT‐1^S113R/+^ mice and H4 cells overexpressing AT‐1 indicate that changes in autophagy induction downstream of AT‐1 are also paralleled by changes in efficiency of the secretory pathway; specifically, reduced AT‐1 activity leads to reduced trafficking of newly synthesized glycoproteins along the secretory pathway while increased AT‐1 activity leads to the opposite effect (Hullinger et al., 2016). To test whether this was indeed the case in AT‐1 sTg mice, we used azide‐modified mannosamine (ManNAz) to label sialic acid‐containing newly synthesized glycoproteins that have successfully trafficked from the ER to the *trans*‐Golgi (Hullinger et al., 2016). The results show a significant increase in the levels of ManNAz incorporation in hepatocytes from AT‐1 sTg animals (Supporting Information Figure S3c). Therefore, when taken together, the combined use of SP‐A53 T syn and ManNAz confirm the conclusion that changes in reticulophagy in AT‐1 sTg mice are closely paralleled by opposite changes in the transport of cargo proteins along the secretory pathway. These results are in line with previous data (Hullinger et al., 2016; Peng & Puglielli, 2016).

In addition to reduced elimination of toxic protein aggregates, a significant block in reticulophagy is expected to cause structural reorganization of the organelle. To determine whether this was indeed the case, we used super‐resolution microscopy, specifically structured illumination microscopy (SIM). Consistent with our prediction, we observed a profound reorganization of the ER in AT‐1 sTg; particularly, we observed expansion of the organelle and enlarged sheet‐like structures (Figure 5c and Supporting Information Figure S3d). Even when comparing similar structures in WT and AT‐1 sTg mice, the transgenic animals displayed a marked membrane proliferation with numerous processes emerging from the ER sheets (Figure 5c; discussed later).

### 2.4 AT‐1 sTg mice display reduced Atg9a‐Fam134b‐LC3β and Atg9a‐Sec62‐LC3β interaction

The role of Atg9a as a potential “sensor” of the acetylation status of the ER has already been described (Pehar et al., 2012; Peng & Puglielli, 2016; Peng et al., 2016, 2014). Thus, the above results are not surprising. However, what remains to be determined is how the acetylation of Atg9a on K359 and K363 within the lumen of the ER can activate the core of the autophagy machinery, which is mainly cytosolic (Klionsky et al., 2016). Interestingly, the above two lysine residues are flanked by two coiled regions that could be involved with protein–protein interactions (Pehar et al., 2012). This might suggest that the acetylation status of Atg9a regulates the interaction with other ER‐luminal or membrane‐bound partners. However, as the core of the autophagy machinery is mainly cytosolic, a membrane‐bound protein is more likely to connect an ER‐luminal event, acetylation of Atg9a, to the autophagy core machinery, specifically LC3β.

To address the above scenario and dissect the mechanism responsible for the block in reticulophagy, we studied levels and ER association of Fam134b, a recently identified key regulator of reticulophagy (Khaminets et al., 2015; Mochida et al., 2015; Rubinsztein, 2015). Interestingly, the ER expansion in AT‐1 sTg mice was accompanied by a marked increase in the number of Fam134b puncta on the ER membrane (Figure 5d,e). The upregulation of Fam134b in AT‐1 sTg mice was also observed when we analyzed mRNA (Figure 5f) and protein levels (Figure 5g,h). Fam134b has recently emerged as a novel regulator of ER‐autophagy, and levels of Fam134b seem to reflect intrinsic dynamics of reticulophagy (Khaminets et al., 2015; Lennemann & Coyne, 2017; Mochida et al., 2015; Nakatogawa & Mochida, 2015; Rubinsztein, 2015). Therefore, increased steady‐state levels of Fam134b in AT‐1 sTg mice (Figure 5d–h) are consistent with the observed ER expansion (Figure 5c and Supporting Information Figure S3d), as they might reflect reduced turnover of the ER‐associated Fam134b protein as well as an attempt of the cell to restore reticulophagy by activating Fam134b translation. Fam134b has a LC3‐interacting region (LIR) on its C‐terminus, which is required for binding to cytosolic LC3β (also referred to as Atg8 in yeast; Khaminets et al., 2015; Mochida et al., 2015; Rubinsztein, 2015). Fam134b‐LC3β interaction is required for efficient induction of reticulophagy (Khaminets et al., 2015; Mochida et al., 2015; Rubinsztein, 2015). Notably, despite the increased levels of Fam134b, we observed a marked reduction in Fam134b‐LC3β co‐localization in AT‐1 sTg vs. WT (Figure 5i,j).

The finding that AT‐1 sTg mice display less Fam134b‐LC3β interaction led us to hypothesize that Atg9a, which acts as a sensor of ER acetylation (Pehar et al., 2012), might engage with Fam134b within the ER membrane. Indeed, immunoprecipitation of Atg9a from the ER membrane was able to pull‐down Fam134b (Figure 5k,l); however, this interaction was markedly reduced in AT‐1 sTg mice (Figure 5k,l) suggesting that the increased acetylation of Atg9a (Figure 5a) impedes functional interaction (see also later). The reduced Atg9a‐Fam134b association in AT‐1 sTg mice was observed despite the fact that we consistently pulled down more Atg9a from the ER of the Tg animals compared to WT littermates (Figure 5k) and that Fam134b is upregulated in the Tg animals (Figure 5g; see also later).

Another important regulator of reticulophagy is Sec62, a member of the ER membrane translocon complex, which regulates import of newly synthesized proteins within the ER (Fumagalli et al., 2016). Like Fam134b, Sec62 also has a LIR domain, which is required for binding to LC3β; increased Sec62‐LC3β interaction promotes reticulophagy and delivery of ER cargo proteins to autolysosomes (Fumagalli et al., 2016). As with Fam134b, SIM imaging revealed a marked reduction in Sec62‐LC3β co‐localization in AT‐1 sTg vs. WT animals (Figure 5m,n). Furthermore, direct biochemical assessment revealed that Atg9a is able to engage Sec62 at the ER membrane; however, the Atg9a‐Sec62 interaction was greatly reduced in the AT‐1 sTg mice (Figure 5o,p). Again, this was observed even though we pulled down more Atg9a from the ER of the Tg animals (Figure 5o). SIM imaging showed that Fam134b‐LC3β and Sec62‐LC3β co‐localization can be observed on the ER membrane (Supporting Information Figure S3e), suggesting that the initial interaction of Fam134b and Sec62 with LC3β can occur on the ER itself prior to the formation of the autophagosome. This process is blocked in AT‐1 sTg mice (Figure 5i–p).

When taken together, the above results suggest that AT‐1 overexpression and increased cytosol‐to‐ER flux of acetyl‐CoA leads to increased acetylation of Atg9a and reduced Atg9a‐Fam134b and Atg9a‐Sec62 interaction within the ER membrane; this prevents interaction with LC3β, thus causing a block in the induction of reticulophagy. Defective reticulophagy is then accompanied by expansion of the organelle. In essence, the acetylation status of Atg9a appears to regulate Fam134b‐LC3β and Sec62‐LC3β interaction and consequent induction of reticulophagy (also discussed later).

The insulin growth factor 1 (Igf‐1) and its receptor (Igf‐1r; Kenyon, 2005; Longo & Finch, 2003; Milman, Huffman, & Barzilai, 2016), as well as the stemness potential of stem cells (Garcia‐Prat et al., 2016; Garcia‐Prat, Sousa‐Victor, & Munoz‐Canoves, 2017), represent already established age‐associated pathways. To determine whether they were mechanistically involved—at least in part—in the AT‐1 sTg phenotype, we analyzed whole tissue activation of Igf‐1r signaling (Supporting Information Figure S4a,b); we also treated cultured MEF with Igf‐1 (Supporting Information Figure S4c,d). However, we did not observe increased Igf‐1r signaling in AT‐1 sTg mice when compared to WT littermates (Supporting Information Figure S4). Similarly, no differences were detected in the percentages of bone marrow Lin^−^Sca^+^c‐Kit^+^ (L^−^S^+^K^+^), multipotent progenitor (MPP; L^−^S^+^K^+^,CD48^+^, CD150^−^), long‐term hematopoietic stem cell (HSC; LT‐HSC; L^−^S^+^K^+^,CD48^−^, CD150^+^), or short‐term HSC (ST‐HSC; L^−^S^+^K^+^,CD48^−^, CD150^−^) populations from bone marrow of WT and AT‐1 sTg mice, suggesting no intrinsic block in stemness potential (Supporting Information Figure S5a). Next, we transplanted WT and AT‐1 sTg bone marrow (CD45.2) into lethally irradiated recipient mice with CD45.1 spleen cells as a supporting cell population and CD45.1 bone marrow cells. Again, no significant differences in contribution of CD45.2 donor cells to bone marrow were observed in WT vs. AT‐1 sTg transplantation (Supporting Information Figure S5b,c).

Many human progeroid syndromes are characterized by nuclear instability where the primary defect is in the architecture and shape of the nuclear envelope; associated animal models reproduce the nuclear instability and mimic the progeroid phenotype (reviewed in Kubben & Misteli, 2017). However, direct assessment of AT‐1 sTg mice did not reveal any morphological aberration of the nucleus (Supporting Information Figure S6).

When taken together, the above results suggest that the reduced Atg9a‐Fam134b‐LC3β and Atg9a‐Sec62‐LC3β interaction, and the consequent block in reticulophagy with changes in efficiency of the secretory pathway are solely responsible for the progeria‐like phenotype of AT‐1 sTg mice.

### 2.5 Inhibition of the ATases rescues the AT‐1 sTg phenotype

**Figure 6:**
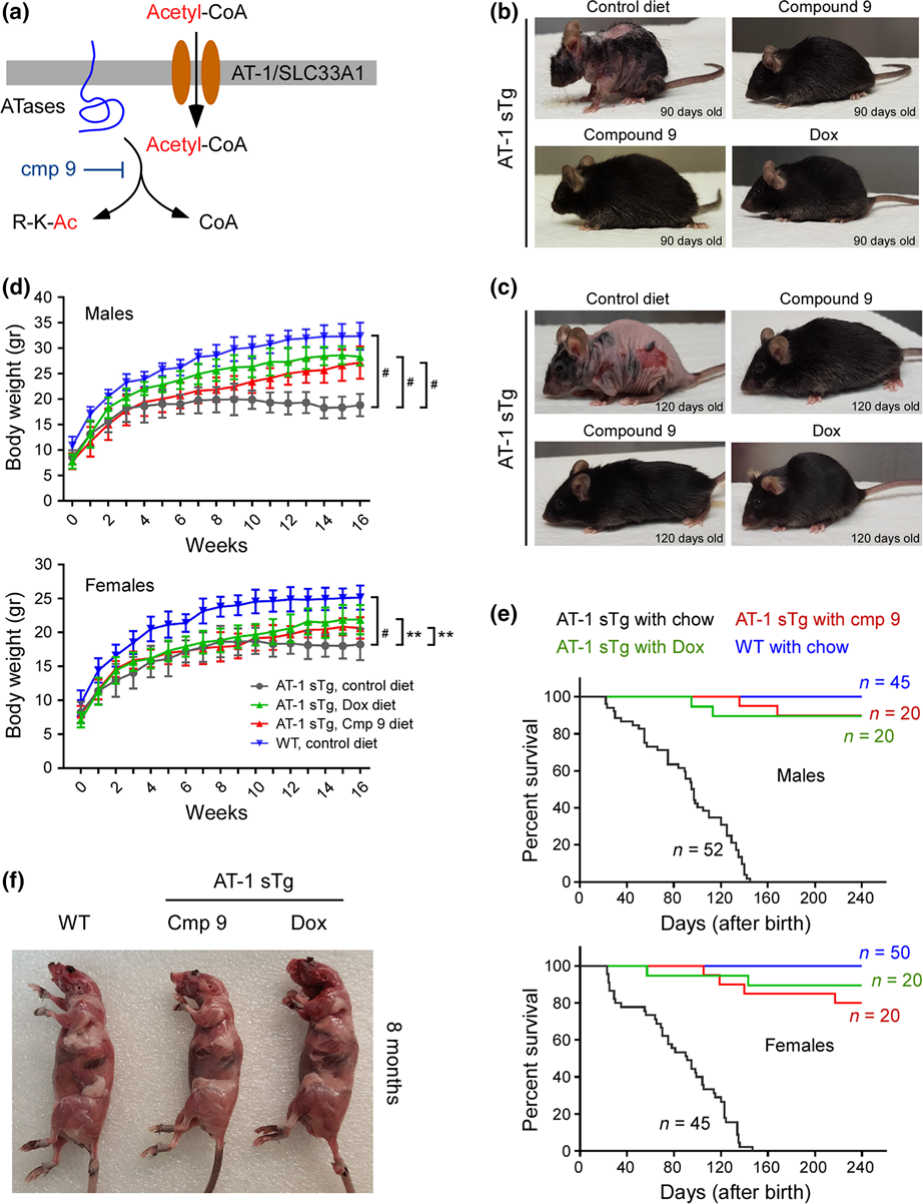
ATase1/ATase2 inhibition rescues the progeria‐like phenotype and lifespan of AT‐1 sTg mice. (a) Schematic view of the ER acetylation machinery with compound 9 acting on the ATases downstream of AT‐1. (b, c) Representative images of AT‐1 sTg mice with and without compound 9 treatment. Dox treatment is shown for comparison. Mice at two different ages are shown. (d) Body weight of male and female AT‐1 sTg mice with and without compound 9 treatment. Dox‐treated AT‐1 sTg mice are shown for comparison (*n* = 16 for all groups). (e) Lifespan of AT‐1 sTg mice with and without compound 9 treatment. Dox‐treated AT‐1 sTg mice are shown for comparison (*p* < 0.0005, all vs. AT‐1 sTg with chow). (f) Postmortem examination of WT and AT‐1 sTg mice treated with compound 9. Dox treatment is shown for comparison. Mice were 8 months old when examined. Bars represent mean ± *SD*. ^**^ *p* < 0.005; ^#^ *p* < 0.0005

In addition to AT‐1, which maintains the supply of acetyl‐CoA to the ER lumen, the acetylation machinery includes two acetyltransferases, ATase1 and ATase2, which use acetyl‐CoA to carry out the reaction of Nε‐lysine acetylation (Ko & Puglielli, 2009). The ATases are ER‐resident membrane proteins and act downstream of AT‐1 (see Figure 6a). We previously reported the identification of ATase1/ATase2‐specific inhibitors (Ding et al., 2012); we also reported the successful use of one of these compounds (6‐chloro‐5H‐benzo[a]phenoxazin‐5‐one; referred to as compound 9) in a mouse model of Alzheimer's disease (Peng et al., 2016). Compound 9 is a powerful inhibitor of both ATases (Ding et al., 2012); it reduces the acetylation of Atg9a, improves the proteostatic functions of the ER, and is orally absorbed (Peng et al., 2016). Therefore, we treated AT‐1 sTg mice with oral formulations of the compound (50 mg kg^−1^ day^−1^; Peng et al., 2016). We argued that if indeed the AT‐1 sTg phenotype is caused by defective reticulophagy and defective elimination of toxic protein aggregates that form within the ER, then restoring the proteostatic functions of the ER by acting downstream of AT‐1 is expected to ameliorate or rescue the phenotype. As an additional control to this study, we added a group of animals fed a Dox‐containing diet to turn off the expression of AT‐1 itself (see Figure 1a). In both cases, compound 9 and Dox were administered at weaning (postnatal day 22–25) when the initial disease phenotypes were already manifested.

Inhibition of the ATase1 and ATase2 by compound 9 rescued the progeria‐like phenotype of AT‐1 sTg mice. Indeed, the animals looked healthy, did not develop skin lesions, alopecia, rectal prolapse, or lordokyphosis (Figure 6b,c), and were able to gain weight as a function of age (Figure 6d). Importantly, compound 9 also rescued the lifespan of the animals (Figure 6e). Postmortem analysis of 8‐month‐old animals confirmed our general assessment. Compound 9‐treated mice had normal fat pads and did not display muscle atrophy (Figure 6f). A complete blood count showed no evidence of anemia, which was paralleled by a normal sized spleen and no evidence of extramedullary erythropoiesis (Supporting Information Figure S7a,b). The lymph nodes were overall normal (Supporting Information Figure S7c); this result was paralleled by normalization of circulating inflammatory molecules (Supporting Information Figure S7d) and the absence of SA‐β‐Gal activation (Supporting Information Figure S7e,f). Compound 9‐treated mice also displayed normal bone mineral density (Supporting Information Figure S7g). Finally, compound 9 was able to rescue the metabolic aspects of the AT‐1 sTg phenotype, including food intake (Supporting Information Figure S7h), circulating levels of glucose (Supporting Information Figure S7i), and cholesterolemia (Supporting Information Figure S7j).

**Figure 7:**
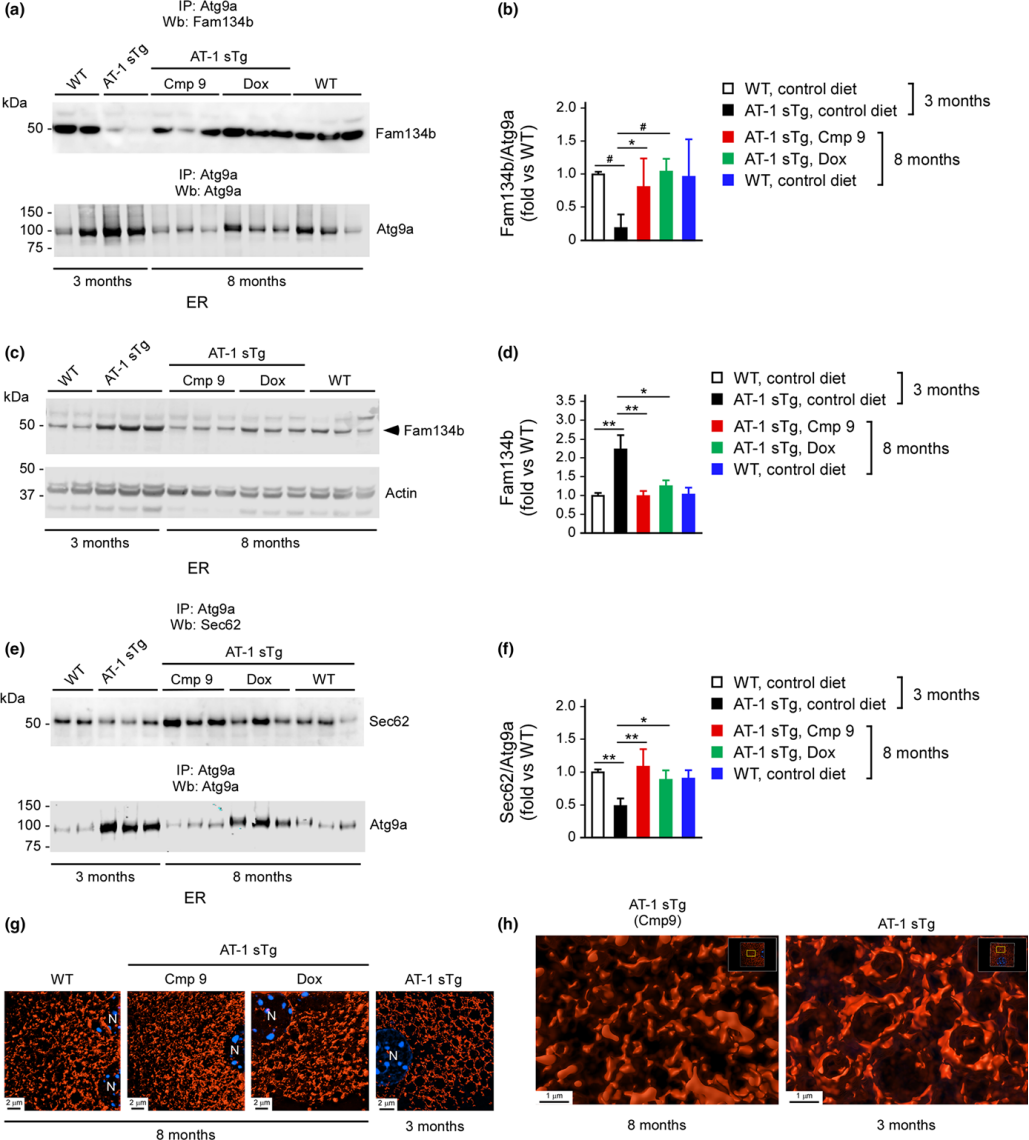
ATase1/ATase2 inhibition restores reticulophagy in AT‐1 sTg mice. (a, b) Western blot showing Atg9a‐Fam134b interaction on the ER membrane of compound 9‐treated AT‐1 sTg mice. Dox‐treated and 3‐month‐old AT‐1 sTg mice are shown for comparison. Representative blots are shown in (a) while quantitation of results is shown in (b) (*n* = 6 for all groups). (c, d) Western blot showing ER levels of Fam134b in compound 9‐treated AT‐1 sTg mice. Dox‐treated and 3‐month‐old AT‐1 sTg mice are shown for comparison. Representative blots are shown in (c) while quantitation of results is shown in (d) (*n* = 6 for all groups). (e, f) Western blot showing Atg9a‐Sec62 interaction on the ER membrane of compound 9‐treated AT‐1 sTg mice. Dox‐treated and 3‐month‐old AT‐1 sTg mice are shown for comparison. Representative blots are shown in (e) while quantitation of results is shown in (f) (*n* = 6 for all groups). (g) Structure illumination microscopy (SIM) of ER in isolated hepatocytes from compound 9‐treated AT‐1 sTg mice. Dox‐treated and 3‐month‐old AT‐1 sTg mice are shown for comparison. (h) High‐magnification images with Imaris‐mediated reconstruction. Bars represent mean ± *SD*. ^*^ *p* < 0.05, ^**^ *p* < 0.005, ^#^ *p* < 0.0005. N: nucleus

Next, we determined Atg9a‐Fam134b interaction as well as levels of Fam134b on ER structures following compound 9 treatment. Again, we observed that AT‐1 sTg mice displayed reduced Atg9a‐Fam134 interaction and increased ER membrane‐associated Fam134b levels; however, both findings were completely normalized by compound 9 treatment (Figure 7a–d). Indeed, compound 9 restored Atg9a‐Fam134b interaction (Figure 7a,b) and normalized Fam134b levels (Figure 7c,d). A similar result was observed with Sec62. In fact, compound 9 restored the Atg9a‐Sec62 interaction at the ER membrane (Figure 7e,f). Finally, compound 9 rescued the membrane expansion and reorganization of the ER observed in AT‐1 sTg mice (Figure 7g,h).

In conclusion, the above results show that restoring the proteostatic functions of the ER by inhibiting ATase1 and ATase2 downstream of AT‐1 rescues the progeria‐like phenotype of AT‐1 sTg mice; they also support the notion that a block in Atg9a‐Fam134b‐LC3b and Atg9a‐Sec62‐LC3β interaction and the consequent defect in reticulophagy are at the basis of the AT‐1 sTg phenotype (Supporting Information Figure S8). Although with some minor differences, the rescue elicited by compound 9 treatment was overall comparable to that of Dox, which in this study acted as our genetic control (see Figures 6 and 7; Supporting Information Figure S7). It is also important to stress that for our postmortem analysis, we used 8‐month‐old animals, which corresponds to approximately twice the lifespan of untreated AT‐1 sTg mice, thus indicating that the protective effects of compound 9 were long lasting.

## 3 DISCUSSION

### 3.1 Increased acetyl‐CoA flux into the ER causes a progeria‐like phenotype

Acetyl‐CoA is a central metabolite that is key to many biochemical and cellular pathways. Acetyl‐CoA also acts as the donor of the acetyl group in all reactions of Nε‐lysine acetylation. Here, we report that increased acetyl‐CoA flux from the cytosol to the ER lumen, as caused by systemic overexpression of human AT‐1 in the mouse, causes a progeria‐like phenotype with metabolic alterations. Segmental progerias typically manifest with severe debilitating symptoms and reduced lifespan. At birth, patients are typically smaller and display facial dysmorphism; as they grow, they develop a complex phenotype that often mimics accelerated forms of pathogenic aging. Segmental progerias include Hutchinson–Gilford, Cokayne, Werner, Bloom, and Rothmund–Thompsons syndromes, among others (Gonzalo et al., 2017; Karikkineth et al., 2017; Kubben & Misteli, 2017; Swahari & Nakamura, 2016).

Almost all human progeroid syndromes are characterized by nuclear and/or genomic instability where the primary defect is in the architecture of the nuclear envelope or in the DNA repairing machinery; associated animal models reproduce the nuclear and genomic instability and mimic the progeroid phenotype (reviewed in (Kubben & Misteli, 2017)). Exceptions to the above are the p53/p44 and the AT‐1 sTg systems. In the case of p53/p44, the primary defect is in the N‐terminal regulatory functions of the p53 protein, which leads to reduced stemness potential of stem cells as well as hyperactivation of IGF‐1R signaling (Campisi, 2004; Lessel et al., 2017; Maier et al., 2004; Pehar, Ko, Li, Scrable, & Puglielli, 2014; Tyner et al., 2002). AT‐1 sTg mice display many features that are in line with classical segmental progerias, such as reduced growth, alopecia, skin lesions, rectal prolapse, osteoporosis, cardiomegaly, muscle atrophy, reduced fertility, and systemic inflammation. Unlike classical progerias, they do not display nuclear/genomic instability, reduced stemness potential of stem cells, or hyperactivation of IGF‐1R signaling. Also in contrast with classical progerias, AT‐1 sTg mice display metabolic‐linked features that are not typically observed in patients with progeria. We contend that the AT‐1 sTg mouse represents the first model of progeria‐like phenotype where the primary defect is in the regulation of intracellular acetyl‐CoA flux, reticulophagy, and proteostatic functions of the ER. In light of the known association between dysfunctional autophagy and age‐associated diseases (Kroemer, 2015; Madeo, Tavernarakis, & Kroemer, 2010; Madeo, Zimmermann, Maiuri, & Kroemer, 2015), we can speculate that AT‐1 sTg mice will offer new mechanistic and therapeutic avenues for several age‐associated diseases.

### 3.2 The progeria‐like phenotype of AT‐1 sTg mice is caused by a block in reticulophagy

Our data suggest that defects in reticulophagy, perhaps accompanied by opposite changes in efficiency of the secretory pathway (Hullinger et al., 2016; Peng & Puglielli, 2016), may causally contribute to the progeria‐like phenotype of the AT‐1 sTg mice. In AT‐1 sTg mice, reticulophagy is linked to the acetylation status of Atg9a indicating that acetyl‐coA flux is a key input for maintenance of ER homeostatic mechanisms (see Supporting Information Figure S8). We have previously shown that Atg9a acts as a sensor of the ER acetylation machinery; indeed, downregulation or expression of hypomorphic AT‐1 lowers levels of acetylation of Atg9a and induces autophagy, while overexpression of AT‐1 leads to increased acetylation of Atg9a and a block in autophagy induction (Pehar et al., 2012; Peng & Puglielli, 2016; Peng et al., 2014). Importantly, gain‐of‐acetylation and loss‐of‐acetylation mutants of Atg9a can recapitulate the events reported above, thus providing mechanistic support (Pehar et al., 2012).

New results reported in this study indicate that functional Atg9a‐Fam134b and Atg9a‐Sec62 association is an initial and essential step for reticulophagy and that these interactions can occur only when Atg9a is not acetylated, highlighting the importance of acetylation on multiple levels in proteostatic control (see Supporting Information Figure S8). Fam134b is an integral ER membrane protein that acts as a “receptor” for reticulophagy (Khaminets et al., 2015; Mochida et al., 2015; Rubinsztein, 2015). It was recently identified in both yeast and mammalian cells (Khaminets et al., 2015; Mochida et al., 2015; Rubinsztein, 2015). Interestingly, Fam134b seems to preferentially localize on ER sheet‐like (rough ER) structures, where the bulk of protein biosynthesis normally occurs, and might act as part of quality control to couple protein biosynthesis to disposal of unfolded/misfolded polypeptides (Nakatogawa & Mochida, 2015). Sec62, an integral member of the ER translocon machinery, also seems to act as an ER‐resident autophagy “receptor” (Fumagalli et al., 2016). Sec62 is also thought to be involved in coupling the insertion of newly synthesized proteins into the ER with the regulatory mechanisms that detect and dispose of unfolded/misfolded polypeptides (Schuck, 2016). Both Fam134b and Sec62 require physical interaction with LC3β to exert their functions (Fumagalli et al., 2016; Khaminets et al., 2015; Mochida et al., 2015; Rubinsztein, 2015; Schuck, 2016). The fact that Atg9a acetylation status directs the formation of Sec62 and Fam134b complexes, sequestering them from the essential binding partner LC3β, suggests coordination of events to influence reticulophagy (see Supporting Information Figure S8). The specific contribution of enhanced efficiency of the secretory pathway to the progeria phenotype of AT‐1 sTg mice remains to be determined.

### 3.3 Biochemical inhibition of the ATases downstream of AT‐1 restores reticulophagy and rescues the progeria‐like phenotype

The impact of pharmacological inhibition of the acetyltransferases, ATase1 and ATase2, is an exciting development and one that may have clinical application. ATase1 and ATase2 act downstream of AT‐1 to acetylate ER cargo proteins (Ko & Puglielli, 2009), regulate the acetylation status of Atg9a, and are essential for the proteostatic functions of the ER acetylation machinery (Ding et al., 2014; Peng et al., 2016). Our study showed that inhibition of the ATases was able to restore the Atg9a‐Fam134b‐LC3β and Atg9a‐Sec62‐LC3β interaction at the ER membrane and rescue the progeria‐like phenotype of AT‐1 sTg mice. Overexpression of AT‐1 in the mouse seems to recapitulate the outcomes of children with duplications of the 3q25.31 locus (containing *AT‐1/SLC33A1*). In this study, treatment with compound 9 was initiated at weaning when the disease manifestations were already developing. This suggests that strategies based on similar targeting might be effective in treating the human disease, where early diagnosis and early treatment could mitigate or even prevent disease manifestations.

While recognizing the intrinsic limitations of a single gene‐directed progeria model, several aspects of the phenotype developed by AT‐1 sTg mice mimic accelerated forms of pathogenic aging. Therefore, the model may provide insights into a range of age‐related diseases and conditions, and studies on ATase1/ATase2 inhibitors might be relevant for chronic diseases linked to proteostatic dysfunction. For example, the entire ER acetylation machinery, AT‐1 (Gomez Ravetti, Rosso, Berretta, & Moscato, 2010; Jonas et al., 2010) and the ATases (Ding et al., 2012), is upregulated in patients with late‐onset Alzheimer's disease, the most common form of age‐associated dementia. Importantly, both haploinsufficiency of AT‐1 and biochemical inhibition of the ATases using compound 9 were able to rescue the Alzheimer's disease‐like phenotype in the mouse (Duran‐Aniotz, Cornejo, & Hetz, 2016; Peng et al., 2016). We are eager to further explore these models and strategies that we believe will have utility in a broader context of aging and age‐related disease.

## 4 CONCLUSION

In conclusion, our study shows that systemic overexpression of AT‐1 in the mouse causes a progeria‐like phenotype with metabolic alterations. Mechanistically, the phenotype is caused by a block in Atg9a‐Fam134b‐LC3β and Atg9a‐Sec62‐LC3β interaction, which prevents the induction of reticulophagy. Our study also shows that restoring the proteostatic functions of the ER, by targeting the ATases downstream of AT‐1, can rescue the mouse phenotype, thus suggesting that ATase1/ATase2 inhibitors might offer translational opportunities for patients with *AT‐1/SLC33A1* duplications and for the mitigation of different age‐associated diseases. Finally, this study sets the foundation for new inquiries into the mechanisms that regulate intracellular acetyl‐CoA flux and availability, and how they can influence disease phenotypes that have not been traditionally viewed as primarily driven by metabolic changes.

## 5 EXPERIMENTAL PROCEDURES

### 5.1 Transgenic animals

pTRE‐AT‐1 Tg mice were described previously (Hullinger et al., 2016). ROSA:LNL:tTA (Gt(ROSA)26Sor^tm1(tTA)Roos^/J; JAX Stock No: 011008) were bred to EIIa‐Cre (B6.FVB‐Tg(EIIa‐cre)C5379Lmgd/J; JAX Stock No: 003724), generating Rosa26:tTA mice which universally express tTA. Rosa26:tTA mice were then crossed with pTRE‐AT‐1 mice to generate ROSA26:tTA;pTRE‐AT‐1 (referred to as AT‐1 sTg) mice. Genotyping from tail DNA was performed using the following primers: AT‐1 forward (5′‐AAT CTG GGA AAC TGG CCT TCT‐3′), AT‐1 reverse (5′‐TAT TAC CGC CTT TGA GTG AGC TGA‐3′), Rosa forward (5′‐AAA GTC GCT CTG AGT TGT TAT‐3′), and Rosa reverse (5′‐GCG AAG AGT TTG TCC TCA ACC‐3′). Both males and females were studied. Wild‐type (WT) littermates were used as controls throughout our study. Unless specified, living AT‐1 sTg mice were studied at the age of approx. 3 months.

The rodent diet with Compound 9 was manufactured by Bio‐Serv. The food with doxycycline (200 mg/kg) was purchased from Bio‐Serv. The same diet without Compound 9 or doxycycline served as the control diet.

All animal experiments were carried out in accordance with the NIH Guide for the Care and Use of Laboratory Animals and were approved by the Institutional Animal Care and Use Committee of the University of Wisconsin‐Madison and the Madison Veterans Administration Hospital.

### 5.2 Cell cultures

Mouse embryonic fibroblasts (MEFs) from wild‐type and AT‐1 sTg mice were prepared as described previously (Peng et al., 2014). Further details are in Supporting Information.

### 5.3 Hepatocyte isolation

Hepatocytes were isolated and analyzed as described in Supporting Information.

### 5.4 Protein extraction, western blotting, and immunoprecipitation

Protein extraction, western blotting, and immunoprecipitation techniques are described in Supporting Information.

### 5.5 Dot blots

Dot blot analysis was performed as described in Supporting Information.

### 5.6 Real‐time PCR

Real‐time PCR was performed as described before (Jonas et al., 2010). Further details are in Supporting Information.

### 5.7 Histology and bone histomorphometry

Histology and bone histomorphometry were performed as described in Supporting Information.

### 5.8 Faxitron radiography and dual‐energy X‐ray absorptiometry

Dual‐energy X‐ray absorptiometry analysis was conducted as described in Supporting Information.

### 5.9 Whole blood, serum, and plasma analytes

Blood, serum, and plasma analytes were determined as in Supporting Information.

### 5.10 Blood and bone marrow smear examination

Fresh whole blood or bone marrow samples were smeared as in Supporting Information.

### 5.11 Flow cytometry

Flow cytometry was performed as in Supporting Information.

### 5.12 Erythroid progenitor assays

Erythroid progenitor assays were performed as in Supporting Information.

### 5.13 Bone marrow transplantation

Bone marrow transplantation experiments were performed as described previously (Wang et al., 2011). Further details are in Supporting Information.

### 5.14 Senescence‐associated β‐galactosidase staining

Senescence β‐galactosidase staining was performed as in Supporting Information.

### 5.15 OGTT, glucose, insulin, and glucagon assays

OGTT, glucose, insulin, and glucagon assays were performed as in Supporting Information.

### 5.16 Echocardiography

Transthoracic echocardiography was performed as described previously (Harris et al., 2002). Further details are in Supporting Information.

### 5.17 Trafficking of newly synthetized glycoproteins

Quantification of trafficking glycoproteins along the secretory pathway was performed as previously described (Hullinger et al., 2016). Further details are in Supporting Information.

### 5.18 Statistics

Data analysis was performed using graphpad instat 3.06 statistical software (GraphPad Software Inc.). Data are expressed as mean ± standard deviation (*SD*). Comparison of the means was performed using Student's *t*‐test or one‐way ANOVA followed by Tukey–Kramer multiple comparisons test. For lifespan assessment, data were analyzed with the Kaplan–Meier lifespan test and log‐rank test using graphpad prism version 7.03 (GraphPad Software). Differences were declared statistically significant if *p* < 0.05.

## CONFLICT OF INTEREST

DWL has received funding from, and is a scientific advisory board member of, Delos Pharmaceuticals, which seeks to develop novel, selective mTOR inhibitors for the treatment of various diseases. DWL's spouse is an employee of DaVita Clinical Research. All others authors have no conflict of interests to disclose.

## author contribution

YP, SLS, VCB, IAD, KJH, GK, KLS, RS, and SIAA performed experiments and all authors analyzed data. EHB, JZ, MPK, ADA, TAH, EK‐G, DWL, RMA, and LP provided critical advice for the experiments. LP designed the overall study and wrote the manuscript with input from all authors.
