# Supplementary material for: Increased transport of acetyl‐CoA into the endoplasmic reticulum causes a progeria‐like phenotype
Source: Aging Cell. 2018 Jul 27;17(5):e12820. doi: 10.1111/acel.12820 (PMC6156544; doi:10.1111/acel.12820)
Supplement: Supplementary file 1 [file ACEL-17-e12820-s001.pdf]

SUPPORTING INFORMATION

Peng et al., "Increased transport of acetyl-CoA into the endoplasmic reticulum causes a progeria-like phenotype".

CONTENTS:

- Experimental Procedures
- References for Experimental Procedures
- Figures S1-S8

EXPERIMENTAL PROCEDURES

Cell cultures

Mouse embryonic fibroblasts (MEFs) from wild-type and AT-1 sTg mice were prepared as described previously (Peng *et al.* 2014). MEFs were maintained in Dulbecco's modified Eagle medium supplemented with 10% fetal bovine serum (FBS) and 1% penicillin/streptomycin/glutamine solution (Mediatech). MEFs were serum-starved for 20 h and then subjected to stimulation with mouse insulin-like growth factor 1 (IGF-1) (cat. no. I8779, Sigma) for 30 min. For transfection, MEFs were transfected with Nucleofector II using the Basic Fibroblast Nucleofector Kit (Lonza). Cells were harvested 48 h later for Western blot. MEF proliferation was measured by Cyquant proliferation assay kit (ThermoFisher Scientific) at the indicated time points.

Hepatocyte isolation

Animals were anesthetized by intraperitoneal injection of approximately 400 mg/kg tribromoethanol (20 mg/ml in PBS). The abdominal cavity was opened and a catheter was inserted into the inferior vena cava for initial perfusion with approximately 100 ml of perfusion buffer (HBSS, 59 mM HEPES, 0.6 mM EGTA; pH 7.5). The portal vein was cut immediately after starting the perfusion. The liver was then perfused with approximately 20 ml of collagenase buffer (HBSS, 0.25 mg/ml of collagenase type IV (Sigman-Aldrich C5138), 7 mM $CaCl_2$, 53 mM HEPES, pH 7.5), removed from the animal, and agitated in perfusion buffer to dissociate hepatocytes. After passing through a 70 µm mesh, hepatocytes were centrifuged (300 rpm, 5 min), re-suspended in washing media (M199), centrifuged again (300 rpm, 5 min), re-suspended in plating media (1% BSA, 1% Pen strep, 1% glutamax, and 10% FBS in M199), and plated (100,000 hepatocytes) on collagen coated (GC-12, Neuvitro) cover slips.

Cultured hepatocytes were transfected with a plasmid carrying ER3-mCherry fusion protein, KDEL, and ER signal peptide (Gift from Michael Davidson; Addgene Plasmid #55041). Transfected hepatocytes were fixed with paraformaldehyde (4%, 15710, Electron Microscopy Sciences), followed by permeabilization with 0.1% Triton^{TM} -X100 (Roche Applied Science) for 5 minutes and incubation in blocking buffer (10% BSA, 5% goat serum in PBS) for one hour. All antibodies were diluted in antibody dilution buffer (1% BSA, 5% goat serum in PBS) and stained with FAM134B (PA542647, ThermoFisher Scientific), LC3B (sc-271625, Santa Cruz biotechnology), and/or Sec62 (ab140644, Abcam) for one hour. Cells were then washed three time in PBS and incubated with secondary antibodies Alexa Fluor488 (A-11034) and Alexa Fluor647 (A-21463); nuclei were stained with DAPI (62248, ThermoFisher Scientific). Cells were washed three time in PBS; prolong diamond antifade mountant (P36965, ThermoFisher

Scientific) was used to mount the cover slips. For imaging, Nikon-N-SIM (structured illumination microscopy) was used and data were analyzed by Imaris image analysis software (Bitplane).

Protein extraction, Western blotting, and immunoprecipitation

Protein extracts were prepared in GTIP buffer (10 mM Tris, pH 7.6, 2 mM EDTA, 0.15 M NaCl) supplemented with 1% Triton™ X-100 (Roche Applied Science), 0.25% Nonidet P-40 (Roche Applied Science), complete protein inhibitor mixture (Roche Applied Science), and phosphatase inhibitors (mixture set I and set II; Calbiochem).

Detergent-soluble and -insoluble fractions were prepared as described (Gan *et al.* 2012; Peng *et al.* 2016). Briefly, cells were lysed in lysis buffer (50 mM Tris-HCl, pH 7.4, 150 mM NaCl, 2 mM EDTA, 1 mM dithiothreitol), complete with protease inhibitors (Roche Applied Science) and 1% Triton™ X-100 (Buffer A), followed by centrifugation at 100,000 *g* for 30 min at 4 °C. Supernatants were recovered as Triton-soluble fractions. Pellets were washed with Buffer A three times, then resuspended in lysis buffer containing Buffer A, 1% sodium dodecyl sulfate (SDS), and 0.5% sodium deoxycholate. After sonication and a brief spin down, the lysates were recovered as Triton-insoluble (SDS-soluble) fractions.

The ER isolation was prepared with a commercial ER Enrichment kit (Novus Biologicals), according to manufacturer's protocol. Briefly, 0.5 gram liver tissue were homogenized in isosmotic homogenization buffer using a Dounce Teflon homogenizer. The homogenized tissue was centrifuged at 1,000 *g* for 10 min at 4 °C and then again at 12,000 *g* for 15 min at 4 °C. Finally, the supernatants were centrifuged at 90,000 *g* for 60 min at 4 °C to obtain total ER fraction. The ER fractions were subjected to Western blot analysis or immunoprecipitation.

Protein concentration was measured by the bicinchoninic acid method (Pierce). Protein electrophoresis was performed on a NuPAGE® system using 4–12% Bis-Tris gels (Invitrogen). The following primary antibodies were used: anti-IGF1R (1:1000; Cell Signaling Technology; 3018), anti-phospho-IGF1R (1:1000; Cell Signaling Technology; 3024), anti-AKT (1:1000; Cell Signaling Technology; 9272), anti-phospho-AKT (1:1000; Cell Signaling Technology; 9271), anti-PTEN (1:1000; Cell Signaling Technology, clone 26H9; 9556), anti-phospho-PTEN (1:1000; Cell Signaling Technology; 9554), anti-SLC33A1/AT-1 (1:1000; Bio-Rad, clone 3A4; MCA3612Z), anti-p16 (1:1000; Abcam; ab189034), anti-ATG9A (1:1000; Abcam, clone EPR2450(2); ab108338), anti-Alpha-synuclein (1:1000; Abcam, clone LB 509; ab27766), anti-FAM134b (1:4000; Abcam; ab151755), anti-LC3B (1:1000; Abcam; ab192890), anti-Sec62 (1:1000; Abcam; ab140644), and anti-β-actin (1:1000; Cell Signaling Technology; 4967). Blots were visualized with goat anti-rabbit or anti-mouse Alexa Fluor® 680-conjugated or Alexa Fluor® 800-conjugated secondary antibodies on infrared imaging (LICOR Odyssey Infrared Imaging System; LI-COR Biosciences).

Immunoprecipitation was performed on protein extracts (1,000 µg) from crude endoplasmic reticulum fractions. Immunoprecipitation was performed using anti-acetylated lysine (1:50; Cell Signaling Technology; 9441), or anti-ATG9A (1:50; Abcam, clone EPR2450(2); ab108338) antibodies and BioMag protein A magnetic particles (Polysciences, Inc.), as described previously (Pehar *et al.* 2012).

Dot blots

Fractions enriched in extracellular (EC), intracellular (IC), membrane-associated (MB), or formic acid-soluble (FA) proteins were prepared as previously described (Lesne *et al.* 2006). Briefly, liver tissue was homogenized in EC buffer (50 mM Tris-HCl, pH 7.6; 150 mM NaCl; 2 mM EDTA; 0.01% (w/v) SDS; and 0.01% (v/v) NP-40) with inhibitors. After centrifugation at 13,200 rpm for 90 min at 4 °C, supernatants containing extracellular proteins were collected. Pellets were then homogenized in IC buffer (50 mM Tris-HCl, pH 7.6; 150 mM NaCl; and 0.1% (v/v) Triton™ X-100; with inhibitors) and re-centrifuged. Resulting IC supernatants were collected, and the remaining pellets were nutated (15 min, 4 °C) in MB buffer (50 mM Tris-HCl, pH 7.4; 150 mM NaCl; 1 mM EGTA; 3% (w/v) SDS; 1% (w/v) deoxycholate; and 0.5% (v/v) Triton™ X-100; with inhibitors). Samples were then centrifuged at 13,200 rpm for 90 min at 4 °C and MB supernatants were collected. To obtain the FA fraction, remaining pellets were homogenized in 40% formic acid and agitated at room temperature for 30 min at 1,400 rpm. Tris (1M, Sigma-Aldrich) was then added to the samples, which were resuspended and centrifuged at 13,200 rpm for 90 min at 4 °C. Resulting supernatants containing formic acid-soluble proteins were collected, centrifuged for 20 min at 13,200 rpm (4 °C), and vacuum concentrated.

One microgram of liver extract enriched in either extracellular, intracellular, membrane-associated, or formic acid-soluble proteins was diluted in PBS to a total volume of 3 µL, then dotted onto a nitrocellulose membrane (Bio-Rad) and allowed to dry. Once dry, membranes were rinsed twice in TBS and incubated for one hour at room temperature in 5% BSA (Sigma-Aldrich) in TBST. Membranes were then incubated overnight at 4 °C in 5% BSA in TBST, washed four times in TBST, and incubated for one hour at room temperature in TBST with Alexa Fluor® conjugated anti-mouse secondary antibody (LICOR Biosciences). After secondary incubation, membranes were washed four additional times in TBST, rinsed once in TBS, then imaged using the LICOR Odyssey Infrared Imaging System. Fluorescent signal for each dot was quantified using OptiQuant (Packard Cyclone, Perkin-Elmer Life Sciences, Inc.).

Real-time PCR

Real-time PCR was performed as described before (Jonas *et al.* 2010). Gene expression levels were normalized against GAPDH levels and expressed as percent of control. The cycling parameters were as follows: 95 °C, 10 s; 55 °C, 10s; 72 °C, 15 s, for a maximum of 45 cycles. Controls without reverse

transcription were included in each assay. Specific primers used are: p21 forward (5′-GTG ATT GCG ATG CGC TCA TG-3'), p21 reverse (5′-TCT CTT GCA GAA GAC CAA TC-3'), FAM134B forward (5'- GGA CTG ACA ATG GGA CCT TC-3'), FAM134B reverse (5'-CTT GGT CTG TGA GCA CTA TCC-3'), GAPDH forward (5′- AGG TCG GTG TGA ACG GAT TTG-3'), and GAPDH reverse (5′-TGT AGA CCA TGT AGT TGA GGT CA-3').

Histology and bone histomorphometry

Hematoxylin and eosin staining was done as described before (Pehar *et al.* 2010; Peng *et al.* 2014). Briefly, tissues were fixed in 10% neutral buffered formalin and embedded in paraffin. Bone tissue was decalcified using a decalcifier solution (Decalcifier I, Leica Biosystems) and embedded in paraffin. Paraffin-embedded tissues were sectioned at 5 microns and stained with hematoxylin and eosin. Hematoxylin and eosin stained slides were examined with a Zeiss Axiovert 200 inverted microscope.

The undecalcified bone histomophometry was performed as described below. Femurs dissected from 3 month-old mice were fixed in 70% ethanol and embedded in prepolymerized polymethyl methacrylate embedding media. The embedded bones were cut at 100 microns using a Saw Microtome (SP1600, Leica Systems). The sections were then subjected to modified Goldner's trichrome staining.

Faxitron radiography and dual-energy X-ray absorptiometry (DEXA)

Bones were fixed in 70% ethanol and soft tissue was removed from the fixed bones. Radiography was performed using a Hewlett Packard Faxitron X-ray system (24 KV for 1.3 min, model 43855A; Hewlett Packard, McMinnville, OR). Bone mineral density (BMD) and total body fat mass were determined using the PIXI-mus small animal dual-energy X-ray absorptiometry (DEXA) device (Lunar) following standard manufacturer protocols. Calibrations were performed with a phantom of known density, and quality assurance measurements were performed prior to BMD measurements.

Whole blood, serum and plasma analytes

Blood was collected transcardially from mice with an insulin syringe. Hematologic parameters were measured on a HemaVet complete blood count (CBC) instrument. For serum, blood was allowed to clot on ice for 15 min, then centrifuged at 1,000 *g* for 10 min at 4 °C and the supernatant was collected. For plasma, blood was collected in BD Microtainer® tubes with K_2EDTA and allowed to clot on ice for 15 min. Following centrifugation at 1,000 *g* for 10 min at 4 °C, the supernatant was collected. Ferritin and iron levels were assayed in serum using Mouse Ferritin ELISA Kit (FTL) (Abcam) and Iron Assay Kit (Abcam), respectively according to manufacturer's instructions. The lipid serum profile was performed by

the UW-Clinical Laboratory. Inflammatory molecules were determined in plasma by Ampersand Biosciences.

Blood and bone marrow smear examination

Fresh whole blood or bone marrow samples were smeared uniformly across a glass slide using another glass slide at a 30 degree smearing angle. The smeared glass slides were then air-dried and stained with PROTOCOL™ Hema 3™ Stain kit (ThermoFisher Scientific) according to manufacturer's instructions. The Hema 3™ stained slides were finally examined with a Zeiss Axiovert 200 inverted microscope.

Flow Cytometry

Bone marrow from WT or AT-1 sTg mice were dissociated, resuspended in PBS with 2% fetal bovine serum (FBS) and passed through a 35 µm nylon filter to obtain single-cell suspensions before antibody staining. All antibodies from eBioscience unless otherwise stated. Analysis of BM LSKs ($Lin^{-}Kit^{+}Sca1^{+}$), MPPs ($Lin^{-}Kit^{+}Sca1^{+}CD48^{-}CD150^{-}$), and HSCs ($Lin^{-}Kit^{+}Sca1^{+}CD48^{-}CD150^{+}$) was conducted using the following antibodies: lineage markers were stained with fluorescein isothiocyanate (FITC)–conjugated B220 (11-0452), CD3 (11-0031), CD4 (11-0041), CD5 (11-0051), CD8 (11-0081), CD41 (11-0411), Gr-1 (11-5931), and TER-119 (11-5921) antibodies. Other surface proteins were detected with PE-Cy7-conjugated CD48 (25-0481), peridinin chlorophyll protein (PerCP)–Cy5.5–conjugated Sca1 (45-5981); PE-conjugated CD150 (12-1502) and APC-conjugated Kit (17-1171) antibodies. Data were collected on a LSRII flow cytometer.

Erythroid progenitor assays

CFU assays were conducted on 8-12 week old WT and AT-1 sTg mice using 0.3 ml spleen suspension at 1 x 10^6 cells/ml mixed with 3 ml of Methocult M3434 (STEMCELL Technologies); 1.1 ml was plated in replicate 35 mm dishes. BFU-E colonies were counted 5 days after culturing. CFU-E colonies were counted 2 days after plating.

Bone marrow transplantation

Adult C57BL/6 recipient mice (CD45.1+; 6–8 weeks old) were lethally irradiated with X-ray (one dose of 8.5 Gy). Bone marrow transplantation experiments were performed as described previously (Wang *et al.* 2011) using 5 × 10^5 WT (n=11) or AT-1 sTg (n=10) Tg bone marrow cells along with the same number of congenic competitor/helper cells in individual lethally irradiated mice. Transplanted cells were monitored by retro-orbital bleeding of the animals at 4-week intervals for 16 weeks after bone

marrow transplantation. Cells were stained with pre-conjugated antibodies specific for the following surface antigens: B220 (11-0452), CD3 (11-0031), CD4 (11-0041), CD8 (11-0081), Gr-1 (11-5931), and TER-119 (11-5921), CD19 (11-0193), IgM (11-5890), CD127 (11-1271) (all from eBiosciences). Stained samples were analyzed on a LSR II flow cytometer (BD Biosciences). The data were analyzed using FlowJo software.

Senescence associated β-galactosidase staining

Primary hepatocytes and cryosections of mouse liver were stained with Senescence β Galactosidase Staining kit (Cell Signaling Technology) according to manufacturer's protocol. Briefly, primary hepatocytes or mouse liver cryosections were fixed with fixation solution for 15 minutes. Fixed sections or cells were then stained with β-galactosidase staining at 37 ºC overnight in a dry incubator without carbon dioxide. Primary hepatocytes were counterstained with DAPI to visualize nuclei. The percentage of senescent cells was expressed as the total number of senescent cells divided by the total number of cells counted using immunofluorescence.

OGTT, Glucose, Insulin and Glucagon assays

Oral glucose tolerance tests (oGTT) were performed on 4-hour fasted (6am-10am) mice. Glucose (2g/kg in water) was administered via oral gavage. Blood was collected by retro-orbital collection prior to glucose administration, and at 5, 15, 30, 60, and 120 minutes following the glucose bolus. Plasma was used to determine glucose and insulin levels. Glucose was measured by the glucose oxidase method using a commercially available kit (TR15221, ThermoFisher Scientific). Insulin was measured by radioimmunoassay (RIA; SRI-13K, Millipore). Glucagon was measured by radioimmunoassay (RIA; GL-32K, Millipore).

Echocardiography

Transthoracic echocardiography was performed using a Visual Sonics 770 ultrasonography with a 30-MHz transducer (RMV 707B) (Visual Sonics, Toronto) as described previously (Harris *et al.* 2002). Mice were lightly anesthetized with isoflurane (1%) and maintained on a heated platform. Two-dimensionally guided M-mode images of the LV and Doppler studies were acquired at the tip of the papillary muscles. LV mass-to-body weight ratio (LV/BW), LV dimension in diastole (LVDd), thickness of the anterior and posterior walls in diastole, and isovolumic relaxation time were recorded. All parameters were measured over at least three consecutive cycles.

Trafficking of newly synthetized glycoproteins

Quantification of trafficking glycoproteins along the secretory pathway was performed as previously described (Hullinger *et al.* 2016). Briefly, nascent glycoproteins in primary hepatocytes were labeled with Click-It™ ManNAz reagent (tetraacetylated N-Azidoacetyl-D-Mannosamine; ThermoFisher Scientific), and visualized with the Click-It™ Cell Reaction Buffer kit (ThermoFisher Scientific). Primary hepatocytes were counterstained with DAPI (62248, ThermoFisher Scientific) to visualize nuclei. The percentage of MaNAz-labeled cells was expressed as the total number of ManNaz-labeled cells divided by the total number of cells counted, using immunofluorescence.

References

Gan, L., Vargas, M.R., Johnson, D.A., Johnson, J.A. (2012). Astrocyte-specific overexpression of Nrf2 delays motor pathology and synuclein aggregation throughout the CNS in the alpha-synuclein mutant (A53T) mouse model. *J Neurosci*. **32**, 17775-17787.

Harris, S.P., Bartley, C.R., Hacker, T.A., McDonald, K.S., Douglas, P.S., Greaser, M.L., … Moss, R.L. (2002). Hypertrophic cardiomyopathy in cardiac myosin binding protein-C knockout mice. *Circ Res*. **90**, 594-601.

Hullinger, R., Li, M., Wang, J., Peng, Y., Dowell, J.A., Bomba-Warczak, E., … Puglielli, L. (2016). Increased expression of AT-1/SLC33A1 causes an autistic-like phenotype in mice by affecting dendritic branching and spine formation. *J Exp Med*. **213**, 1267-1284.

Jonas, M.C., Pehar, M., Puglielli, L. (2010). AT-1 is the ER membrane acetyl-CoA transporter and is essential for cell viability. *J Cell Sci*. **123**, 3378-3388.

Lesne, S., Koh, M.T., Kotilinek, L., Kayed, R., Glabe, C.G., Yang, A., … Ashe, K.H. (2006). A specific amyloid-beta protein assembly in the brain impairs memory. *Nature*. **440**, 352-357.

Pehar, M., Jonas, M.C., Hare, T.M., Puglielli, L. (2012). SLC33A1/AT-1 protein regulates the induction of autophagy downstream of IRE1/XBP1 pathway. *J Biol Chem*. **287**, 29921-29930.

Pehar, M., O'Riordan, K.J., Burns-Cusato, M., Andrzejewski, M.E., del Alcazar, C.G., Burger, C., … Puglielli, L. (2010). Altered longevity-assurance activity of p53:p44 in the mouse causes memory loss, neurodegeneration and premature death. *Aging Cell*. **9**, 174-190.

Peng, Y., Kim, M.J., Hullinger, R., O'Riordan, K.J., Burger, C., Pehar, M., Puglielli, L. (2016). Improved proteostasis in the secretory pathway rescues Alzheimer's disease in the mouse. *Brain*. **139**, 937-952.

Peng, Y., Li, M., Clarkson, B.D., Pehar, M., Lao, P.J., Hillmer, A.T., … Puglielli, L. (2014). Deficient Import of Acetyl-CoA into the ER Lumen Causes Neurodegeneration and Propensity to Infections, Inflammation, and Cancer. *J Neurosci*. **34**, 6772-6789.

Wang, J., Liu, Y., Li, Z., Wang, Z., Tan, L.X., Ryu, M.J., … Zhang, J. (2011). Endogenous oncogenic Nras mutation initiates hematopoietic malignancies in a dose- and cell type-dependent manner. *Blood*. **118**, 368-379.

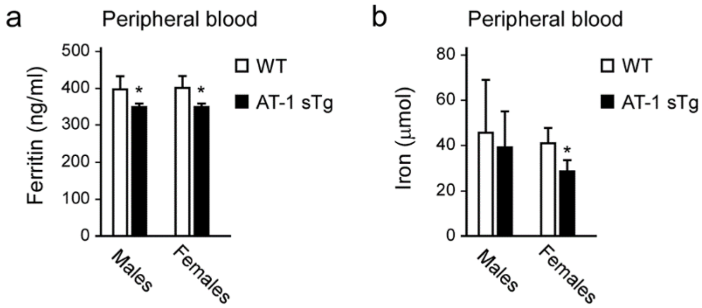

Figure S1. AT-1 sTg mice display reduced serum levels of ferritin and iron. (a, b) Serum levels of ferritin (a) and iron (b) in WT and AT-1 sTg mice (males, n=3; females, n=3). Error bars indicate SD. $^{*}p<0.05$.

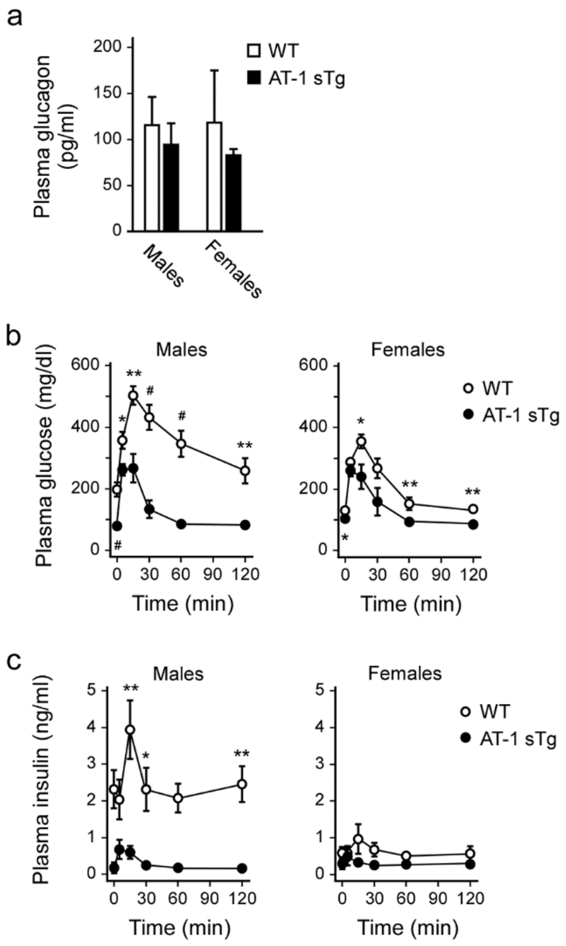

Figure S2. AT-1 sTg mice display reduced levels of circulating glucose and insulin. (a) Fasting levels of glucagon in plasma (males, n=5; females, n=5). (b, c) Oral glucose tolerance test (OGTT) in WT and AT-1 sTg mice (males, n=5; females, n=5). Plasma glucose (b) and insulin (c) levels are shown. Error bars indicate SD. $^{*}p<0.05$, $^{**}p<0.005$, $^{\#}p<0.0005$.

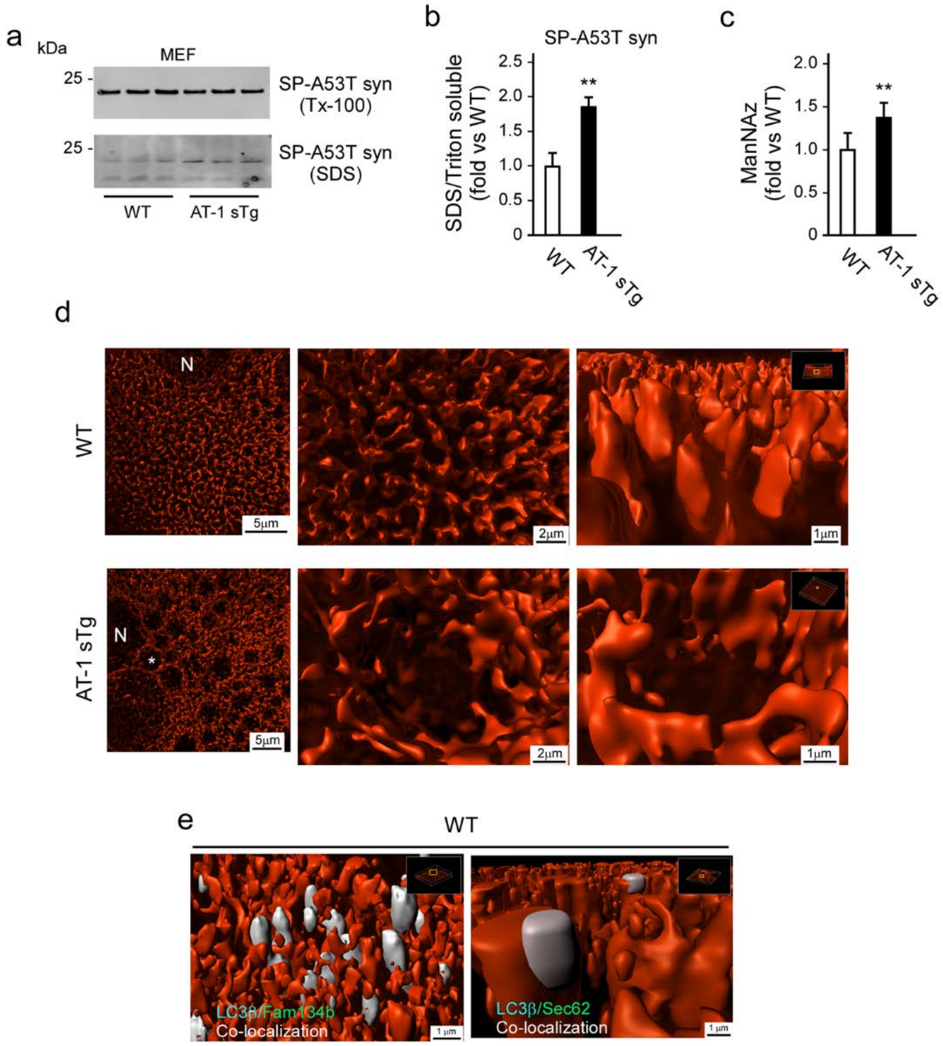

Figure S3. AT-1 sTg mice display structural reorganization and expansion of the ER. (a, b) Western blot showing Triton X-100 soluble (Tx-100) and insoluble (SDS) levels of SP-A53T syn expressed in WT and AT-1 sTg MEF. Representative blots are shown in (a) while quantitation of results is shown in (b) (WT, n=3; AT-1 sTg, n=3). (c) Incorporation of ManNAz into sialic acid-containing glycoproteins in WT and AT-1 sTg MEF (WT, n=6; AT-1 sTg, n=6). (d) Structure illumination microscopy (SIM) of ER in isolated hepatocytes from WT and AT-1 sTg mice. N, nucleus; asterisk (*) indicates a typical sheet-like structure. (e) Super-resolution Imaris-mediated visualization of Fam134b/LC3β (*left*) and Sec62/LC3β (right) puncta (white/grey) on ER (red) of isolated hepatocytes. Error bars indicate SD. $^{**}p<0.005$.

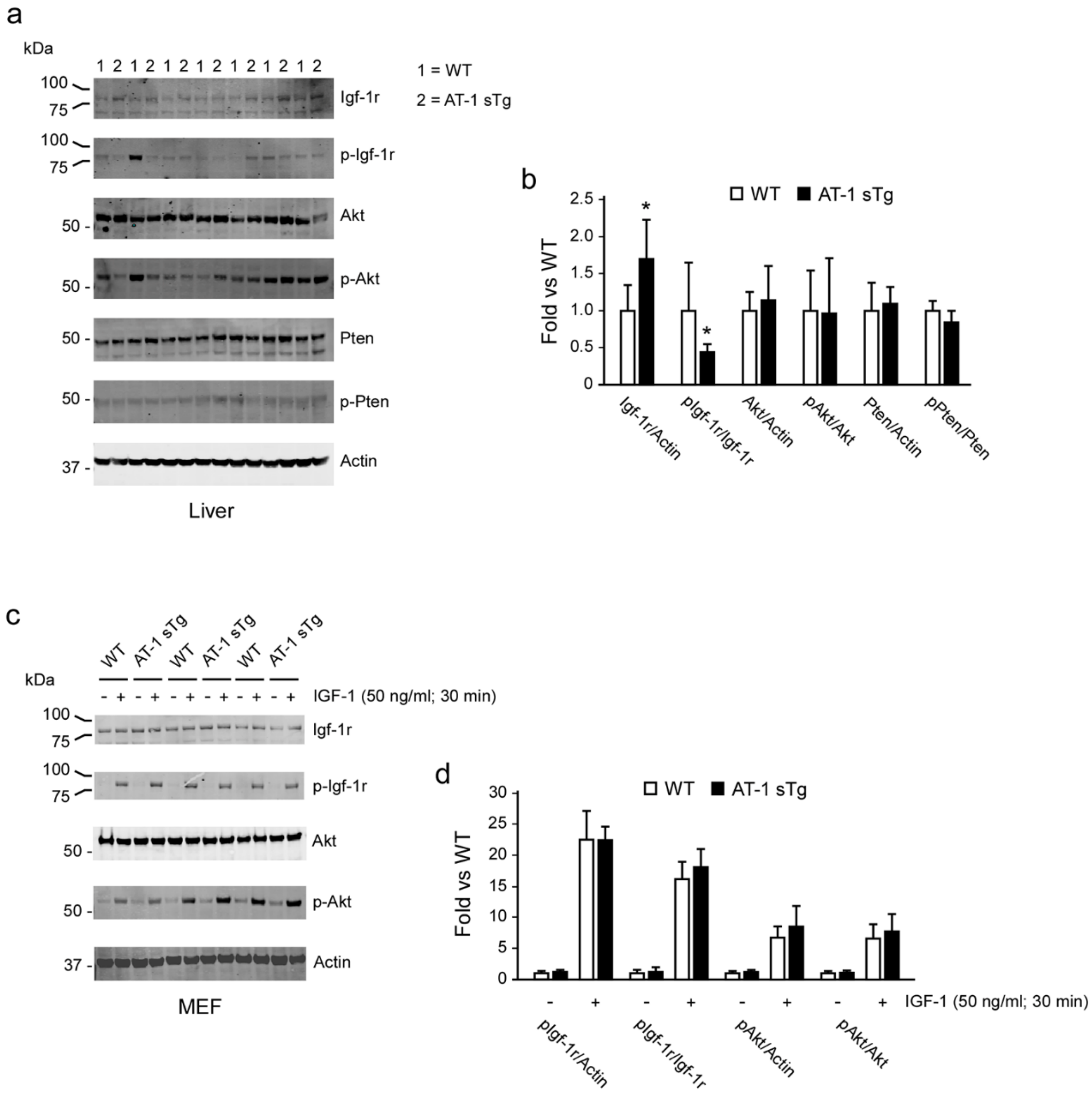

Figure S4. AT-1 overexpression does not affect IGF-1R signaling. (a, b) Western blot of Igf-1r and downstream targets in WT and AT-1 sTg mice (liver). Representative images (a) and quantification of results (b; WT, n=7; AT-1 sTg, n=7) are shown. (c, d) Western blot showing Igf-1r and Akt phosphorylation in MEF from WT and AT-1 sTg mice treated with IGF-1. Representative images (c) and quantification of results (d; n=3 different MEF per group) are shown. Error bars indicate SD. $^{*}p<0.05$.

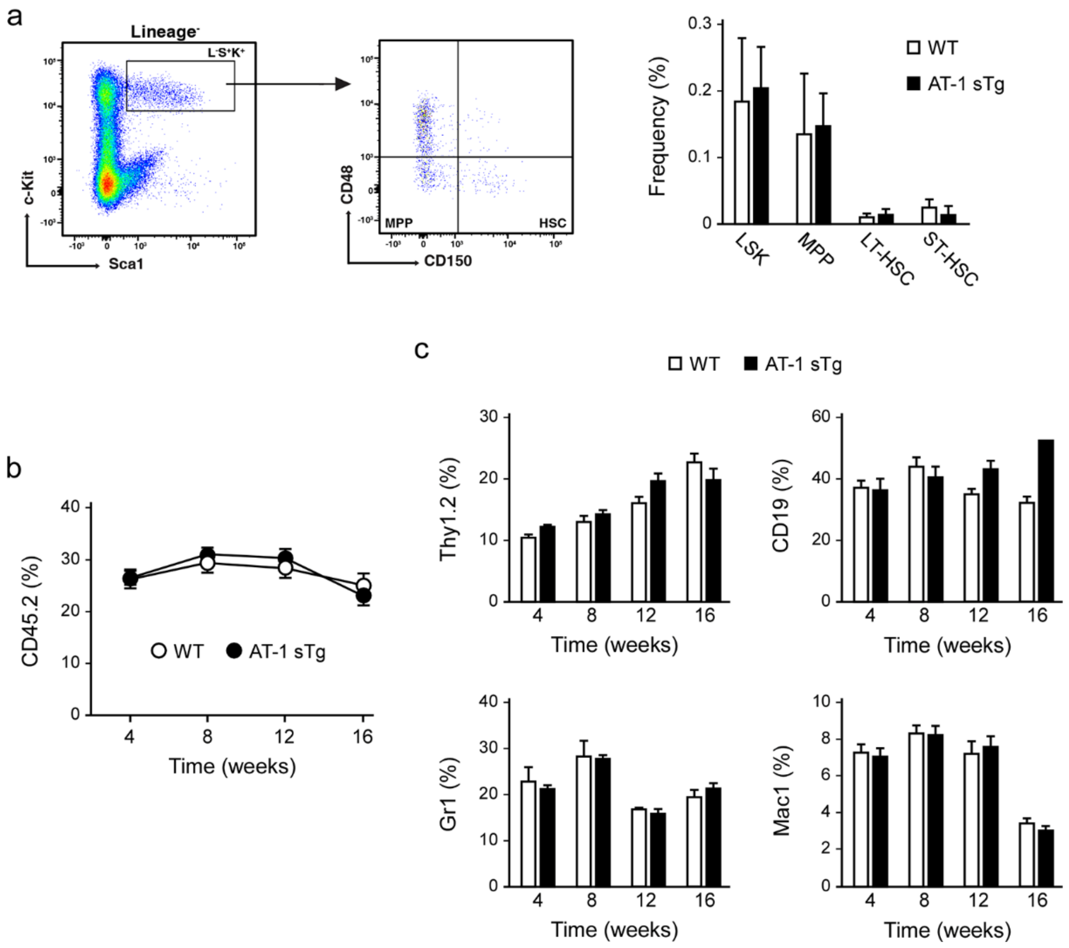

Figure S5. AT-1 Tg overexpression does not impact hematopoietic stem cell levels or function. (a) Representative flow cytometric plots (*left*) and quantitation (*right*) of Lin$^-$Sca-1$^+$c-kit$^+$ (L$^-$S$^+$K$^+$) cells, multipotent progenitor (MPP; L$^-$S$^+$K$^+$,CD48$^+$, CD150$^-$), long-term HSC (LT-HSC; L$^-$S$^+$K$^+$,CD48$^-$, CD150$^+$), and short-term HSC (ST-HSC; L$^-$S$^+$K$^+$,CD48$^-$, CD150$^-$) populations from bone marrow of WT and AT-1 sTg mice (n=6 per group). (b) Contribution from WT (N=11) or AT-1 sTg (n=10) bone marrow in a competitive transplantation assay. (c) Contribution of WT (n=11) and AT-1 sTg (n=10) donor-derived CD45.2 cells to peripheral myeloid cells (Mac1$^+$), granulocytes (Gr1$^+$), B cells (CD19$^+$) and T cells (Thy1.2$^+$). Error bars indicate SD.

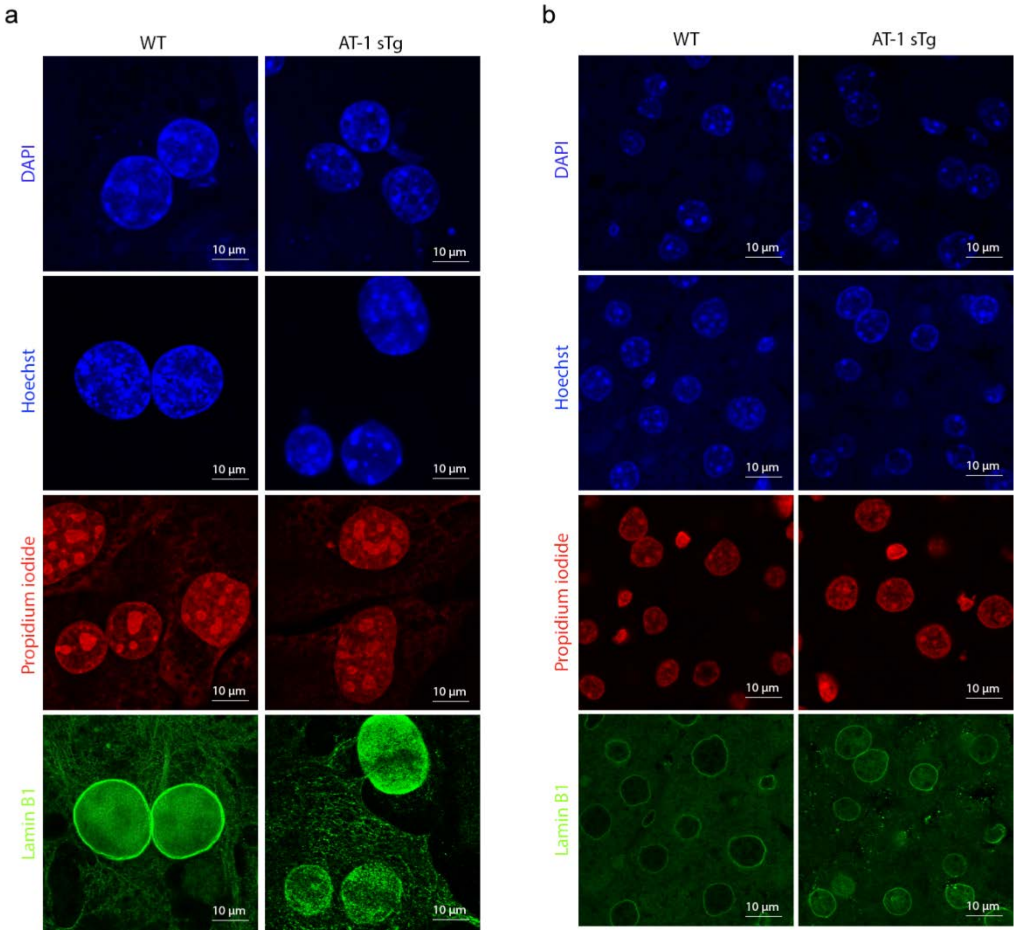

Figure S6. AT-1 Tg overexpression does not affect nuclear morphology. (a) Isolated hepatocytes. (b) Liver.

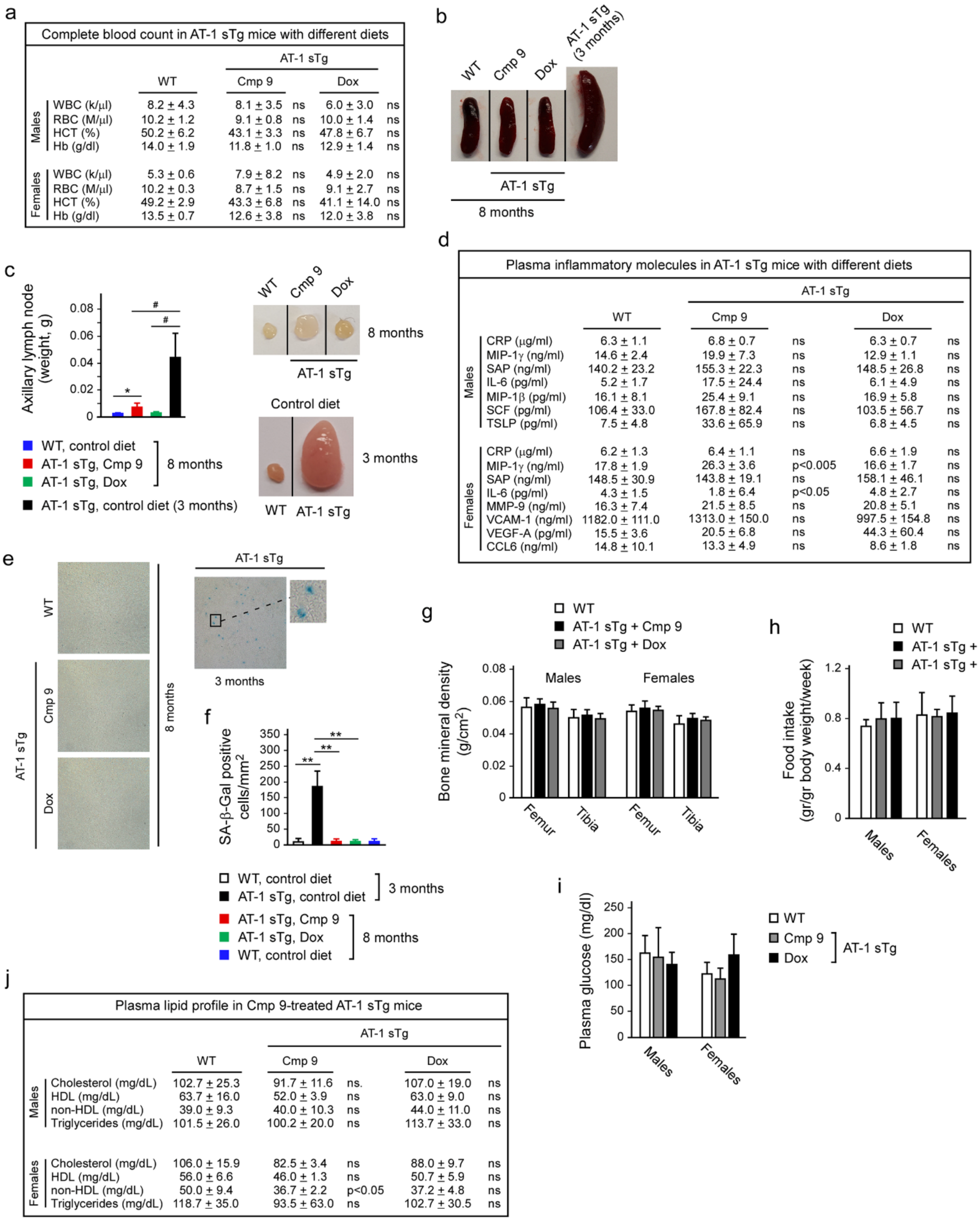

a Complete blood count in AT-1 sTg mice with different diets

		WT	AT-1 sTg: Cmp 9		AT-1 sTg: Dox	
Males	WBC (k/μl)	8.2 ± 4.3	8.1 ± 3.5	ns	6.0 ± 3.0	ns
	RBC (M/μl)	10.2 ± 1.2	9.1 ± 0.8	ns	10.0 ± 1.4	ns
	HCT (%)	50.2 ± 6.2	43.1 ± 3.3	ns	47.8 ± 6.7	ns
	Hb (g/dl)	14.0 ± 1.9	11.8 ± 1.0	ns	12.9 ± 1.4	ns
Females	WBC (k/μl)	5.3 ± 0.6	7.9 ± 8.2	ns	4.9 ± 2.0	ns
	RBC (M/μl)	10.2 ± 0.3	8.7 ± 1.5	ns	9.1 ± 2.7	ns
	HCT (%)	49.2 ± 2.9	43.3 ± 6.8	ns	41.1 ± 14.0	ns
	Hb (g/dl)	13.5 ± 0.7	12.6 ± 3.8	ns	12.0 ± 3.8	ns

d Plasma inflammatory molecules in AT-1 sTg mice with different diets

		WT	AT-1 sTg: Cmp 9		AT-1 sTg: Dox	
Males	CRP (μg/ml)	6.3 ± 1.1	6.8 ± 0.7	ns	6.3 ± 0.7	ns
	MIP-1γ (ng/ml)	14.6 ± 2.4	19.9 ± 7.3	ns	12.9 ± 1.1	ns
	SAP (ng/ml)	140.2 ± 23.2	155.3 ± 22.3	ns	148.5 ± 26.8	ns
	IL-6 (pg/ml)	5.2 ± 1.7	17.5 ± 24.4	ns	6.1 ± 4.9	ns
	MIP-1β (pg/ml)	16.1 ± 8.1	25.4 ± 9.1	ns	16.9 ± 5.8	ns
	SCF (pg/ml)	106.4 ± 33.0	167.8 ± 82.4	ns	103.5 ± 56.7	ns
	TSLP (pg/ml)	7.5 ± 4.8	33.6 ± 65.9	ns	6.8 ± 4.5	ns
Females	CRP (μg/ml)	6.2 ± 1.3	6.4 ± 1.1	ns	6.6 ± 1.9	ns
	MIP-1γ (ng/ml)	17.8 ± 1.9	26.3 ± 3.6	p<0.005	16.6 ± 1.7	ns
	SAP (ng/ml)	148.5 ± 30.9	143.8 ± 19.1	ns	158.1 ± 46.1	ns
	IL-6 (pg/ml)	4.3 ± 1.5	1.8 ± 6.4	p<0.05	4.8 ± 2.7	ns
	MMP-9 (ng/ml)	16.3 ± 7.4	21.5 ± 8.5	ns	20.8 ± 5.1	ns
	VCAM-1 (ng/ml)	1182.0 ± 111.0	1313.0 ± 150.0	ns	997.5 ± 154.8	ns
	VEGF-A (pg/ml)	15.5 ± 3.6	20.5 ± 6.8	ns	44.3 ± 60.4	ns
	CCL6 (ng/ml)	14.8 ± 10.1	13.3 ± 4.9	ns	8.6 ± 1.8	ns

j Plasma lipid profile in Cmp 9-treated AT-1 sTg mice

		WT	AT-1 sTg: Cmp 9		AT-1 sTg: Dox	
Males	Cholesterol (mg/dL)	102.7 ± 25.3	91.7 ± 11.6	ns.	107.0 ± 19.0	ns
	HDL (mg/dL)	63.7 ± 16.0	52.0 ± 3.9	ns	63.0 ± 9.0	ns
	non-HDL (mg/dL)	39.0 ± 9.3	40.0 ± 10.3	ns	44.0 ± 11.0	ns
	Triglycerides (mg/dL)	101.5 ± 26.0	100.2 ± 20.0	ns	113.7 ± 33.0	ns
Females	Cholesterol (mg/dL)	106.0 ± 15.9	82.5 ± 3.4	ns	88.0 ± 9.7	ns
	HDL (mg/dL)	56.0 ± 6.6	46.0 ± 1.3	ns	50.7 ± 5.9	ns
	non-HDL (mg/dL)	50.0 ± 9.4	36.7 ± 2.2	p<0.05	37.2 ± 4.8	ns
	Triglycerides (mg/dL)	118.7 ± 35.0	93.5 ± 63.0	ns	102.7 ± 30.5	ns

Figure S7. ATase1/ATase2 inhibition rescues all major features of the AT-1 sTg progeria-like phenotype. (a) Hematologic parameters of WT and compound 9-treated AT-1 sTg mice; Dox-treated animals are shown for comparison (males, n=5; females, n=5; Age=8 months). *WBC*, white blood cells;

RBC, red blood cells; *HCT*, hematocrit; *Hb*, hemoglobin. (b) Representative images of whole spleen from WT and compound 9-treated AT-1 sTg mice. Dox-treated and 3-month old AT-1 sTg mice are shown for comparison. (c) Weight of axillary lymph nodes in WT and compound 9-treated AT-1 sTg mice. Dox-treated and 3-month old AT-1 sTg mice are shown for comparison (n=6 for all groups). (d) Plasma inflammatory markers (males, n=6; females, n=6; Age=8 months). Statistical significance *vs* WT is shown. (e, f) SA-β-Gal staining in liver of compound 9-treated AT-1 sTg mice. Dox-treated and 3-month old AT-1 sTg mice are shown for comparison. Representative images are shown in (e) while quantitation of results is shown in (f; males, n=3; females, n=3; Age=8 months). (g) Bone mineral density of WT and compound 9-treated AT-1 sTg mice. Dox treatment is shown for comparison (males, n=5; females, n=5; Age=8 months). (h) Food intake of WT and compound 9-treated AT-1 sTg mice. Dox-treated mice are shown for comparison (males, n=5; females, n=5). (i) Fasting levels of glucose in plasma (males, n=5; females, n=5; Age=8 months). (j) Plasma lipid profile in WT and AT-1 sTg mice (males, n=5; females, n=5; Age=8 months). Dox-treated mice are shown for comparison. Bars represent mean $\pm$ SD. $^{*}p<0.05$, $^{**}p<0.005$, $^{\#}p<0.0005$.

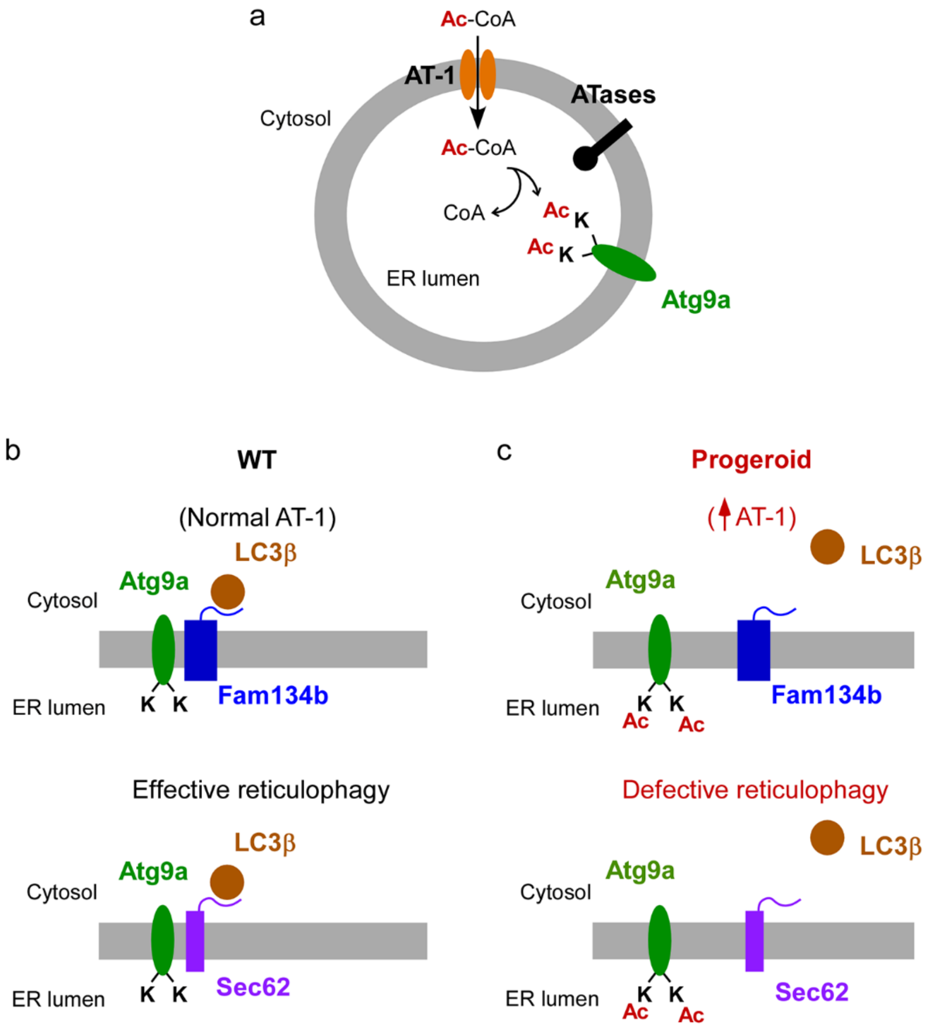

Figure S8. Schematic model summarizing the results of this study. (a) AT-1 regulates the transport of acetyl-CoA (Ac-CoA) from the cytosol to the ER lumen. In the ER lumen, Ac-CoA is used by ATase1 and ATase2 to acetylate ER cargo and resident proteins. The acetylation of Atg9a on K359 and K363 within the ER lumen regulates the induction of reticulophagy. (b) When not acetylated, Atg9a is able to interact with Fam134b and Sec62 thus allowing further engagement of LC3β. The formation of the Atg9a-Fam134b-LC3β and Atg9a-Sec62-LC3β complexes induces reticulophagy. (c) The acetylation of Atg9a prevents interaction with Fam134b and Sec62 thus preventing engagement of LC3β and induction of autophagy. The increased expression of AT-1 in AT-1 sTg mice leads to increased influx of acetyl-CoA into the ER lumen and hyper-acetylation of Atg9a (see a). This prevents the formation of Atg9a-Fam134b-LC3β and Atg9a-Sec62-LC3β complexes and blocks the induction of reticulophagy, thus

leading to the progeria-like phenotype (see c). The phenotype is rescued by an ATase1/ATase2 inhibitor, which prevents hyper-acetylation of Atg9a.
